# Control of human gene expression: High abundance of divergent transcription in genes containing both INR and BRE elements in the core promoter

**DOI:** 10.1371/journal.pone.0202927

**Published:** 2018-08-23

**Authors:** Jay C. Brown

**Affiliations:** Department of Microbiology, Immunology and Cancer Biology, University of Virginia School of Medicine, Charlottesville, Virginia, United States of America; University of Naples Federico II, ITALY

## Abstract

**Background:**

DNA sequence elements in the core promoter can play a central role in regulation of gene expression. Core elements (e.g. INR and TATA box) are located within ~50bp of the transcription start site and both upstream and downstream elements are known. Although all can affect the level of gene expression, their mechanism of action has yet to be fully defined. The studies described here are focused on two core promoter elements, INR and BRE, in the human genome. The locations of the two elements were determined in a large number of human promoters and the results were interpreted in terms of overall promoter function.

**Results:**

A total of 13,406 promoters were collected from the reference version of the human genome and found to contain 62,891 INR sequences and 32,290 BRE. An INR sequence was found in the core region of 1231 (9.2%) promoters and a BRE in 2592 (19.3%); 121 promoters (0.9%) have both INR and BRE elements. Counts support the view that most human promoters lack an INR or BRE element in the core promoter. Further analysis was carried out with the aligned aggregate of promoters from each chromosome. The results showed distinct INR distributions in separate chromosome groups indicating a degree of chromosome specificity to the way core promoter elements are deployed in the genome. The rare promoters with both INR and BRE elements were found to be enriched among the genes with divergent transcription. Enrichment raises the possibility that core promoter elements can have a function in chromosome organization as well as in initiation of transcription.

## Introduction

Control of gene expression is central to the functioning of all living organisms. The need to modulate expression of an invariant set of genes is at the center of development, homeostasis and the ability of organisms to adapt to environmental change. It is not surprising therefore that regulation of gene expression is one of the most actively-studied areas in all of biology and that we know so much about it. Although gene expression is controlled at multiple levels, investigators continue to emphasize the first step in the overall process, the transcription of gene DNA into mRNA by DNA-dependent RNA polymerase II (RNAPII). This enzyme and the means to control its activity are found in all three domains of life [1], and it is thought that a thorough understanding of transcription by RNAPII will take us a long way toward understanding the overall landscape of gene expression.

Regulation of RNAPII activity in humans and other higher metazoans is thought to involve two classes of mechanisms called canonical and non-canonical [2–10]. Canonical processes are by far the more thoroughly studied. Expression of a gene is found to be controlled by a set of cis-acting sequence elements recognized by proteins involved in initiation of transcription. Two categories of cis-acting sequences are recognized: (1) core promoter elements such as INR, TATA box and BRE upstream of the transcription start site and DCE, MTE and others downstream [10]; and (2) protein transcription factor binding sites.

Core promoter sequences are found within ~50bp of the transcription start site (TSS) and are recognized by general transcription factors such as TFIID and TFIIB [2, 11]. Together their role is to create a pre-initiation complex (PIC) consisting of RNAPII plus general transcription factors in which RNAPII is poised to begin RNA synthesis at one or a few closely spaced start sites. A synthetic core promoter sequence has been shown to initiate start-site-specific RNA synthesis in vitro [12]. Cryo-electron microscopy has been used to determine the three-dimensional structure of PIC components bound to a synthetic core promoter DNA [13].

Transcription factor sites are quite different from core promoter elements. For instance, transcription factor (TF) sites can be hundreds of base pairs removed from the TSS. Each site is recognized by its own TF, and more than 1000 TF-TF site pairs have been identified in the human genome [14–18]. It is thought that transcription factors influence transcription by attaching to their specific binding site and also to the pre-initiation complex. Binding of physically remote TF’s may require DNA looping to allow TF-PIC contact. The effect of TF binding may be to potentiate or attenuate the extent of RNA synthesis. It is considered that the combination of core promoter elements and transcription factors functioning together may provide the extent of gene regulation required by the biology of most organisms.

Three observations have suggested the need for non-canonical in addition to canonical mechanisms for the regulation of RNAPII transcription. First, in the case of mammalian genomes, the concept of a core promoter is not considered to apply [10]. Instead, transcription start sites are embedded in larger regions 100bp-200bp long found to be enriched in CpG islands and other non-canonical elements [10]. Second, studies of eukaryotic organisms have demonstrated that a very high proportion (~90%) of the genome is transcribed into RNA [19, 20]. Transcription is observed on both DNA strands and in regions of the genome that do not encode proteins as well as those that do. It is reasoned that such a high level of transcription would be observed only if at least some of it were functional. Third, a portion of genes in higher eukaryotes are found to be depleted in one or more of the known core promoter elements and some have none at all. It is argued that such genes require novel mechanisms to drive their expression [10, 21].

INR and BRE core promoter elements are best known for the way they function in canonical promoters. INR has the sequence YYANWYY and is recognized by the general transcription factor TFIID. In canonical promoters INR spans the TSS [2]. Two TFIIB recognition elements (BRE) are found to influence gene expression in canonical promoters. Called BREu and BREd, the two are located 35bp and 20bp, respectively, upstream of the TSS. The roles of INR and BRE elements have not been thoroughly described in non-canonical promoters.

I have been examining core promoter elements in the human genome with the goal of defining their role in non-canonical promoters. The major resource for the study is a database of more than 13,000 human gene promoters, and analysis is focused on INR and BRE elements. Here I report results showing the distribution of the two elements in the aggregate of promoters present in each human chromosome. Further analysis is focused on promoters that contain both INR and BRE elements in the core promoter region.

## Materials and methods

### Promoter database

Promoters were identified by the FirstEF algorithm [22] and downloaded from the 2003 (hg16) version of the reference human genome using the UCSC Genome Browser (https://genome.ucsc.edu/). Downloading was performed one chromosome at a time beginning at the left chromosome end. Promoters were examined individually and accepted for the database only if they were located upstream and near to an annotated, functional gene. Downloaded promoters were all 1000bp in length beginning 570bp upstream from the transcription start site and extending to 430bp downstream. Downstream sequences were included in case analysis revealed a role for them in control of gene expression. Each accepted promoter was downloaded initially into a computer file called the megafile which contains all the promoters downloaded from a single chromosome. The megafile was then split computationally into 1000bp individual promoters with each given a number preceded by “list”. Promoters were downloaded from all 24 human chromosomes, 22 autosomes plus the X and Y chromosomes. Megafile and list files are available online at: (http://www.people.virginia.edu/~akr4xc/Human%20Gene%20Promoters%20-%20Home.html).

### Location of INR and BRE elements

INR and BRE locations were determined beginning with the megafile for each chromosome. Sequences employed for INR and BRE were (C|T)(C|T)A(A|T|G|C)(A|T)(T|C)(T|C) and (G|C)(G|C)(A|G)CGCC, respectively. Otherwise identification of the INR and BRE locations was similar and the method is described below only for INR. The locations of INR sequences were determined by use of a Python script based on re.find beginning with one entire chromosome sequence. The script returned the position of the first nucleotide in each of the INR sequences identified. Depending on the chromosome, a few hundred to a few thousand INR positions were returned. To align the promoter sequences together, all but the right-most three positions of the location number was stripped away using the RIGHT function in Microsoft Excel. The remaining list contained only the position of each INR within its own promoter sequence. A histogram was plotted of the list numbers using the histogram function in SigmaPlot 13.0 and rendered graphically also with SigmaPlot.

The locations of INR and BRE sequences within the core promoter were determined using a similar method to that described above for the entire promoter. In the case of INR, re.find was employed to identify all the promoter INR sequences in a chromosome and Excel RIGHT was used to remove all but the INR position within its own promoter. INR sequences within the core region were then identified using the Excel routine Conditional Formatting>Highlight Cells>Between. As described in the text, -20bp-+10bp with respect to the TSS was used for the core region of INR and -60bp-+10bp was used for BRE. Methods described above were used for computing a histogram and for rendering it graphically.

### Promoters with INR+BRE and multiple BRE sequences

Core promoters containing both INR and BRE elements were identified one chromosome at a time beginning with the list of core promoters containing an INR and the list of BRE-containing core promoters. The two lists were aligned and the list numbers of promoters containing both elements in the core were added to a separate file that ultimately contained the identities of INR+BRE core promoters from the entire database. The same method was used to identify core promoters with two or more BRE elements.

### Genes driven by promoters with both INR and BRE elements in the core region

NCBI BLAST was used to identify the genes driven by the 121 promoters found to have both INR and BRE sequences in the core region. The same method was used to identify the genes driven by the control promoters described below. In all cases, the entire promoter sequence was used to probe the 2013 version of the reference human genome (hg18). A unique gene or gene pair was returned in every case. A transcript was considered to be a part of a divergently-transcribed pair if its start site was within 2000bp of a transcript synthesized in the opposite direction. Not counted were un-transcribed candidate genes and pseudogene transcripts.

The count of divergently-transcribed gene pairs with INR+BRE core promoters was compared to three control counts: (1) 121 genes chosen at random from the human genome; (2) 121 genes chosen at random from those containing an INR, but not a BRE in the core promoter; and (3) 121 genes chosen at random from those with a BRE, but no INR in the core promoter. In each of the three control gene sets, the number of genes from each chromosome was matched to the number present in the INR+BRE set.

## Results

### The promoter database

The main resource for the study was a database of 13,406 human promoters. Promoters were identified by the FirstEF algorithm [22] and downloaded individually from the 2003 version (hg16) of the reference human genome using the UCSC Genome Browser. A promoter was included in the database only if it is in close proximity to the 5’ end of an annotated, functional gene. All downloaded promoters are 1000bp in length including 570bp upstream of the TSS and 430bp downstream. Each promoter was downloaded into a computer file containing all the promoters in a single chromosome. Further information about the database is included in the Materials and Methods section.

### INR and BRE elements in each chromosome

Table 1 shows the number of promoters collected into the file for each chromosome. The average is 559 ± 248 promoters/chromosome with a range of 35 (Y chromosome) to 1107 (chromosome 1). The number of INR and BRE sequences was counted in each chromosome file. Sequences used for INR and BRE counts were, respectively, (C|T)(C|T)A(A|T|G|C)(A|T)(T|C)(T|C) and (G|C)(G|C)(A|G)CGCC [10, 21]. The BRE sequence used corresponds to the upstream of the two recognized BRE elements. The aggregate of all chromosomes showed a total of 62,891 INR elements and 32,290 BRE. As the total number of promoters was 13,406, the counts indicate an average of 4.7 INR and 2.4 BRE elements per promoter examined.

**Table 1:** INR and BRE elements in the promoters of human genes.

Chr	Promoters in database	INR in all promoters	INR in core promoter^a^	BRE in all promoters	BRE in core promoter^b^	INR BRE both in core promoter
1	1107	5135	71	2599	213	9
2	739	3274	89	1865	145	7
3	666	3245	48	1635	146	6
4	470	2928	61	926	64	3
5	533	2504	38	1209	96	1
6	545	3494	85	949	60	1
7	822	3692	69	2080	180	5
8	600	2644	37	1567	126	4
9	707	3208	63	1900	147	7
10	458	2151	34	1230	114	5
11	627	2935	58	1419	108	9
12	814	3957	116	1799	137	14
13	306	1443	25	676	53	2
14	367	1762	33	865	81	7
15	439	1988	43	1072	89	5
16	799	3320	53	2089	164	3
17	706	3086	58	1746	142	5
18	261	1180	25	796	68	4
19	880	4061	87	1937	158	8
20	587	2580	70	1496	120	8
21	147	544	8	432	27	1
22	361	1472	21	1096	83	2
X	430	2143	35	846	63	4
Y	35	145	4	61	8	1
Total	13,406	62,891	1231 (9.2%)	32,290	2592 (19.3%)	121 (0.9%)

^a^ Bp -20-+10 with respect to the TSS.

^b^ Bp -60-+10 with respect to the TSS.

Similar counts were made using just the core promoter regions of each promoter (Table 1). The core promoter was considered to extend from bps -20-+10 in the case of INR and -60-+10 for BRE. The results showed that of the 13,406 promoters sampled, 1231 (9.2%) had an INR sequence in the core promoter and 2592 (19.3%) had a BRE. 121 core promoters (0.9%) were found to have both INR and BRE elements.

**Fig 1:**
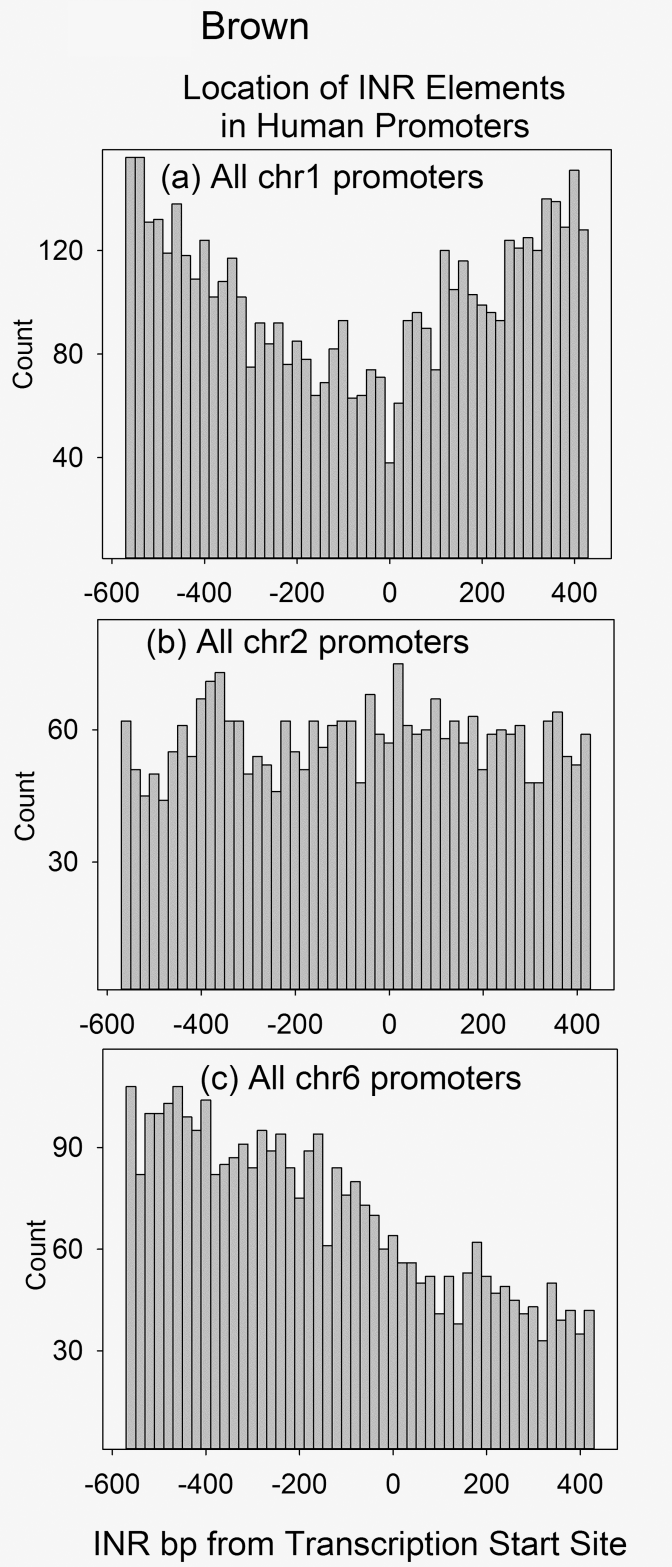
Histograms showing the location of INR elements in the promoters of human chromosomes 1, 2 and 6. In each panel the count shown is the number of INR elements present in a 20bp window of the aggregate file produced by aligning all the database promoters from the same chromosome. Chromosomes were selected to illustrate the V (a), flat (b) and sloped (c) distributions. Histograms for all human chromosomes can be found in S1 Powerpoint.

In view of the small number of INR elements in individual promoters, INR sequences were considered as a group in all the promoters of each chromosome. Hundreds to thousands (depending on the chromosome) of INR locations were therefore considered at once permitting a comprehensive view of core element locations. For each of the 1000 positions in a promoter, the number of INR elements starting at each location was added and the sums were plotted as a histogram (Fig 1). The bar height therefore indicates the number of INR sequences at the same position in all the database promoters of a single chromosome. The results showed three different distributions, V, flat and sloped, that depended on the chromosome examined. The V distribution was the most prevalent occurring in 19 of the 24 human chromosomes (Fig 1A; see also S1 Powerpoint). Here the concentration of INR elements was found to be greatest at the extremes of the compound promoter sequence with a minimum at or very near the position of the TSS. This distribution was unexpected; it was assumed that INR sequences would be concentrated at the TSS where they have been shown to be functional. Instead, only 38 of 1107 chromosome 1 promoters were found to have an INR beginning exactly at the TSS. Similar results were found with other V-distribution chromosomes. The observation raises the question of whether INR elements can be functional if they are located remotely from the TSS.

The flat distribution (Fig 1B) shows little dependence of INR density on location within the 1000bp promoter sequence. Found in 3 of the 24 human chromosomes (chrs 2, 12 and 22), this distribution shows no concentration, depletion or other singularity of INR elements at the TSS. The same is true for the sloped distribution (Fig 1C) found in two chromosomes (4 and 6). Like the V distribution, the flat and sloped distributions were unexpected and raise the question of where INR elements need to be located to be functional.

**Fig 2:**
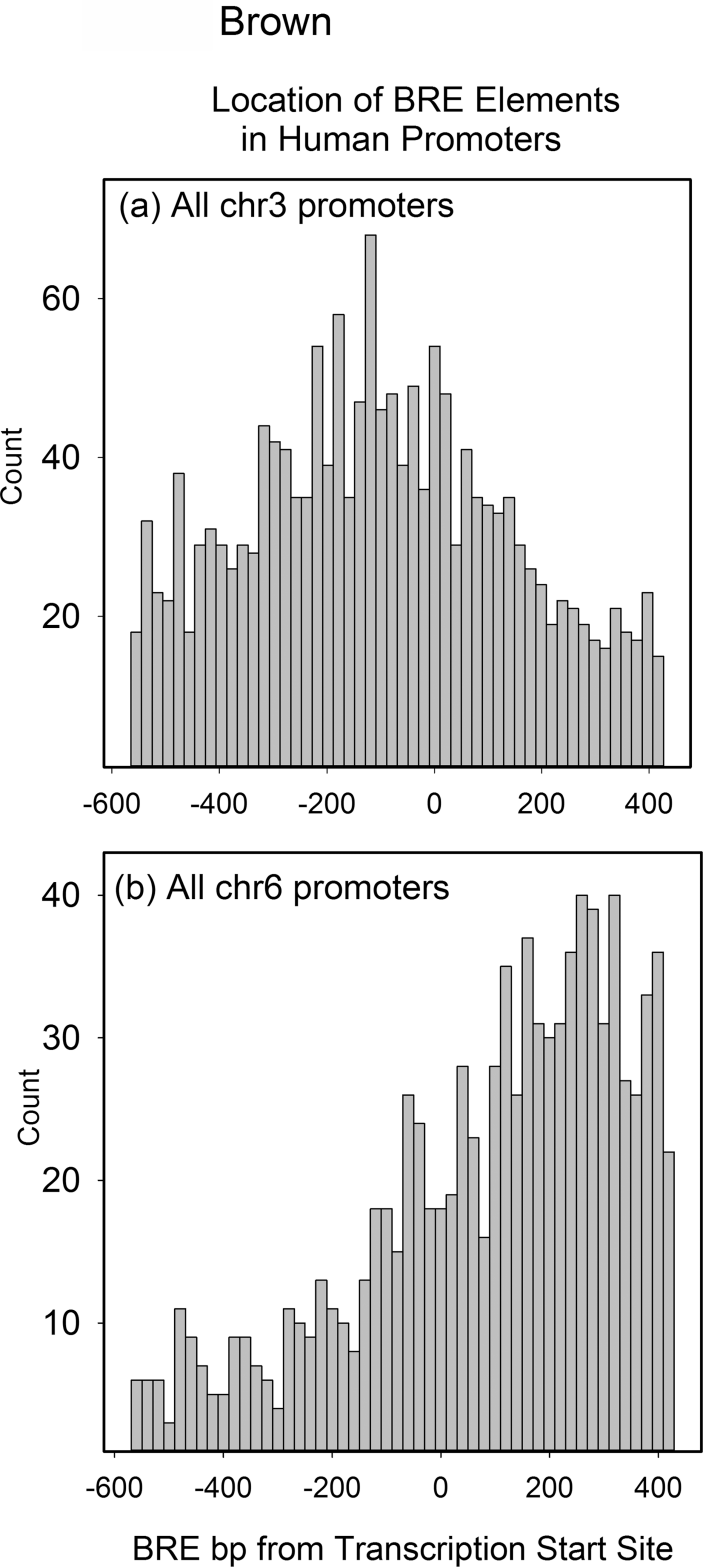
Histograms showing the location of BRE elements in the promoters of human chromosomes 3 and 6. Each count is the number of BRE elements present in a 20bp window of the aggregate file produced by aligning all the database promoters from the same chromosome. Chromosomes were selected to illustrate the central peak (a) and skewed peak (b) distributions. Histograms for all human chromosomes can be found in S2 Powerpoint.

The distribution of BRE elements was examined in the same way described above for INR. Like the results with INR, the results with BRE demonstrated a dependence on chromosome. Two types of distribution were found, central peak and skewed peak distributions (Fig 2A and 2B, respectively; see also S2 Powerpoint). Central peak distributions were the more abundant. They were found in 16 of 22 chromosomes in which the distribution could be classified. Skewed distributions could be skewed to either the upstream or downstream direction of the promoter and were found in chromosomes 4–7, 12 and X. Distributions could not be classified for chromosomes 21 and Y. In central peak distributions the maximum was found to be near but rarely exactly at the position of the TSS (see Fig 2A). Neither BRE distribution showed any feature indicating a singularity at the position of the TSS.

### INR and BRE elements in the core promoter

**Fig 3:**
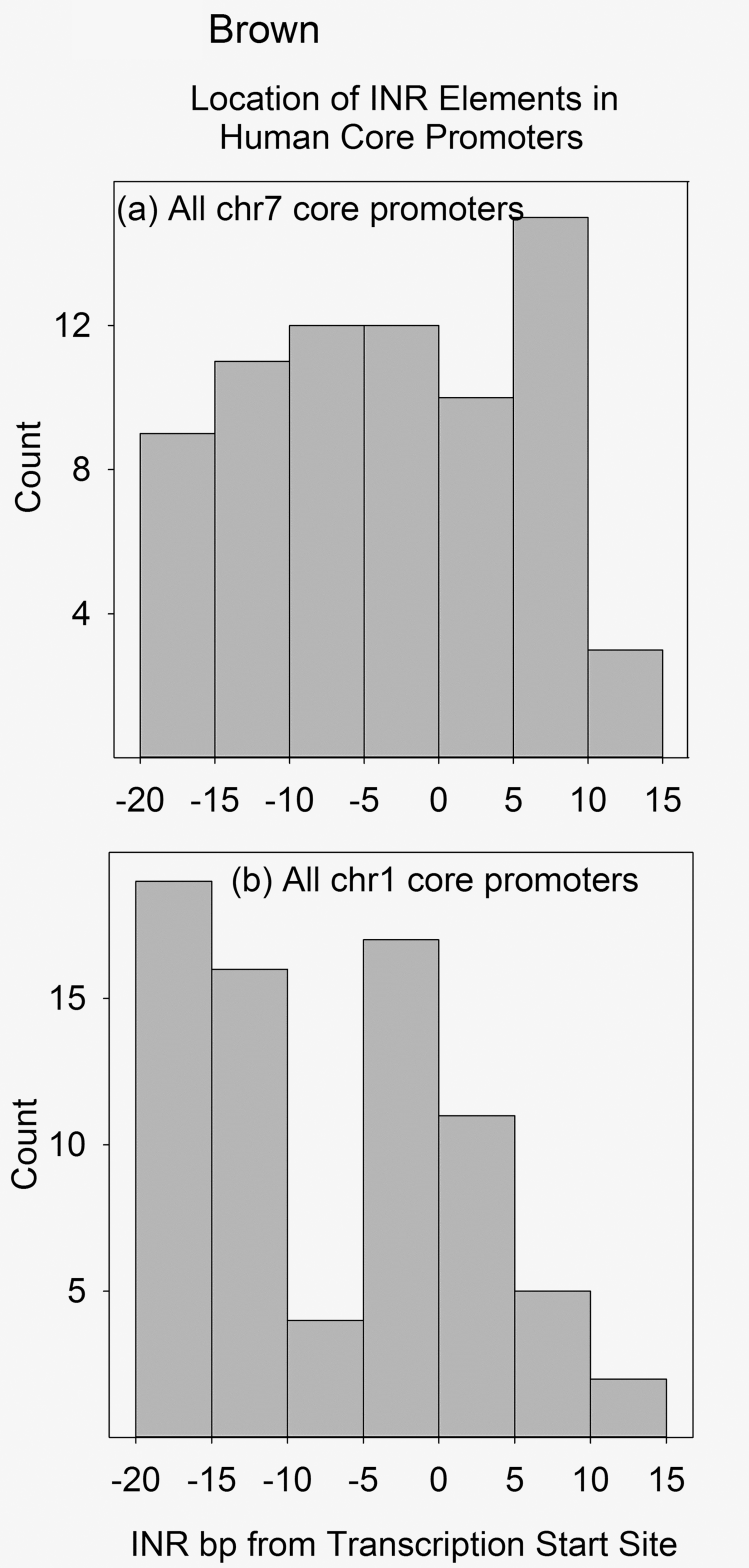
Histograms showing the location of INR elements in the core promoter regions of human chromosomes 7 and 1. Each count is the number of INR elements in a 5bp window of the aggregate file produced by aligning all the database core promoters from the same chromosome. Chromosomes were selected to illustrate the unimodal (a) and bimodal (b) distributions. Histograms for other chromosomes can be found in S3 Powerpoint.

**Fig 4:**
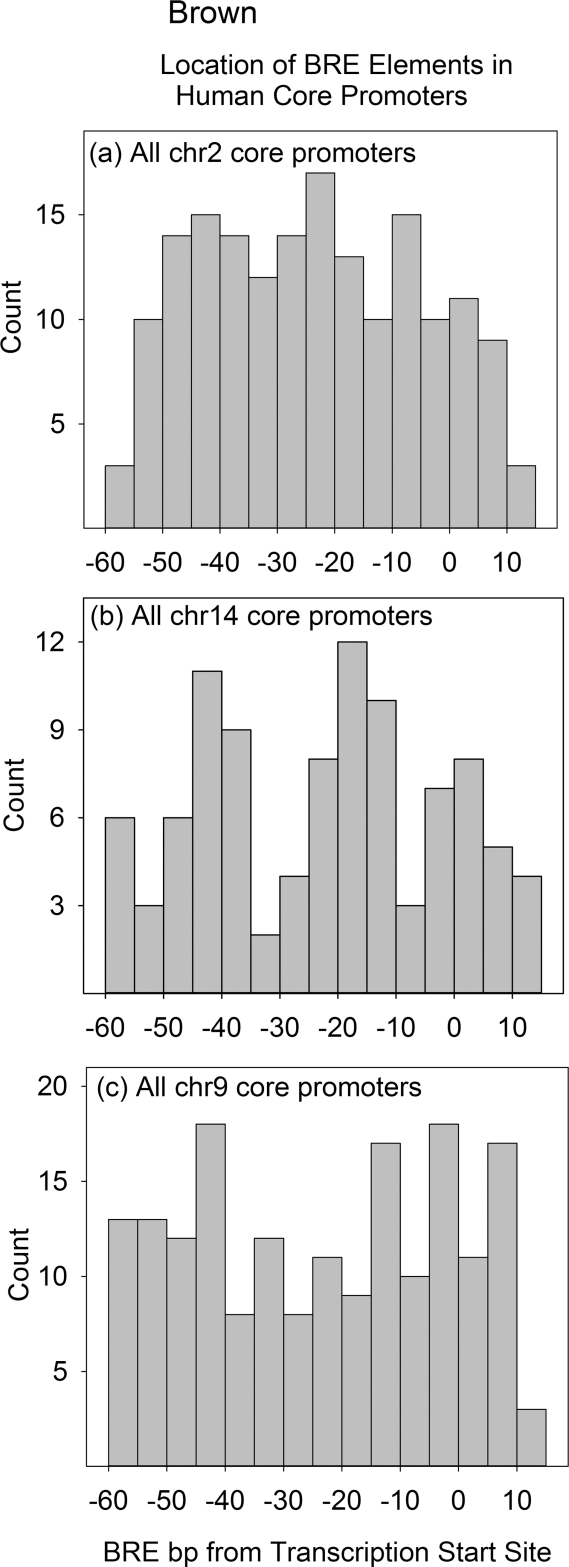
Histograms showing the location of BRE elements in the core promoter regions of human chromosomes 2, 14 and 9. Each count is the number of BRE elements in a 5bp window of the aggregate file produced by aligning all the database core promoters from the same chromosome. Chromosomes were selected to illustrate the flat (a), trimodal (b) and multimodal (c) distributions. Histograms for other chromosomes can be found in S4 Powerpoint.

Because INR and BRE elements are both thought to function primarily within the core promoter, locations of the two elements were examined specifically in the core. Core promoters were considered to extend from -20-+10bp with respect to the TSS in the case of INR and -60-+10bp for BRE. Alignments were created as described above for a set of promoter sequences. INR or BRE elements beginning at each bp in the target region were counted in an entire chromosome and the counts were plotted as a histogram. Representative results are shown in Figs 3 and 4 with the entire datasets available in S3 and S4 Powerpoints.

As in the case of the whole promoter studies, both INR and BRE element core distributions were found to differ in different chromosomes. Two distinct distributions were observed in the case of INR, a unimodal and a bimodal distribution. Examples of the two are shown in Fig 3A and 3B, respectively. In the unimodal distribution the density of INR elements does not vary dramatically across the core promoter length. In contrast, two distinct concentrations are found in the bimodal distribution, a feature not previously reported for INR locations. The unimodal distribution was observed more often than the bimodal. Of 22 chromosomes that could be classified (all but 21 and Y), 16 were unimodal and 6 bimodal. The presence of a bimodal distribution raises the possibility that the two INR classes may have different functions.

Three distributions were recognized in the case of BRE, flat, trimodal and multi-modal (Figs 4A–4C). Little effect of position on BRE density was detected in the flat distribution while three and more than three were observed in tri- and multi-modal distributions, respectively. Neither trimodal nor multimodal BRE distributions have been reported previously. The flat distribution was the one observed most often. Of 22 chromosomes that could be classified (all but 21 and Y), 12 were judged as flat, 6 as trimodal and 4 as multimodal. Evidence of periodicity was observed in both the trimodal and multimodal distributions. BRE concentrations were separated by ~20bp in the case of the trimodal distribution and ~10bp in the case of the multimodal (see Fig 4B and 4C). The existence of discrete concentrations of BRE-TSS distance suggests this separation may affect BRE function. The observed periodicity may be due to oligomeric states of proteins able to recognize BRE sequences (see Discussion).

### INR-BRE and BRE-BRE separations

**Fig 5:**
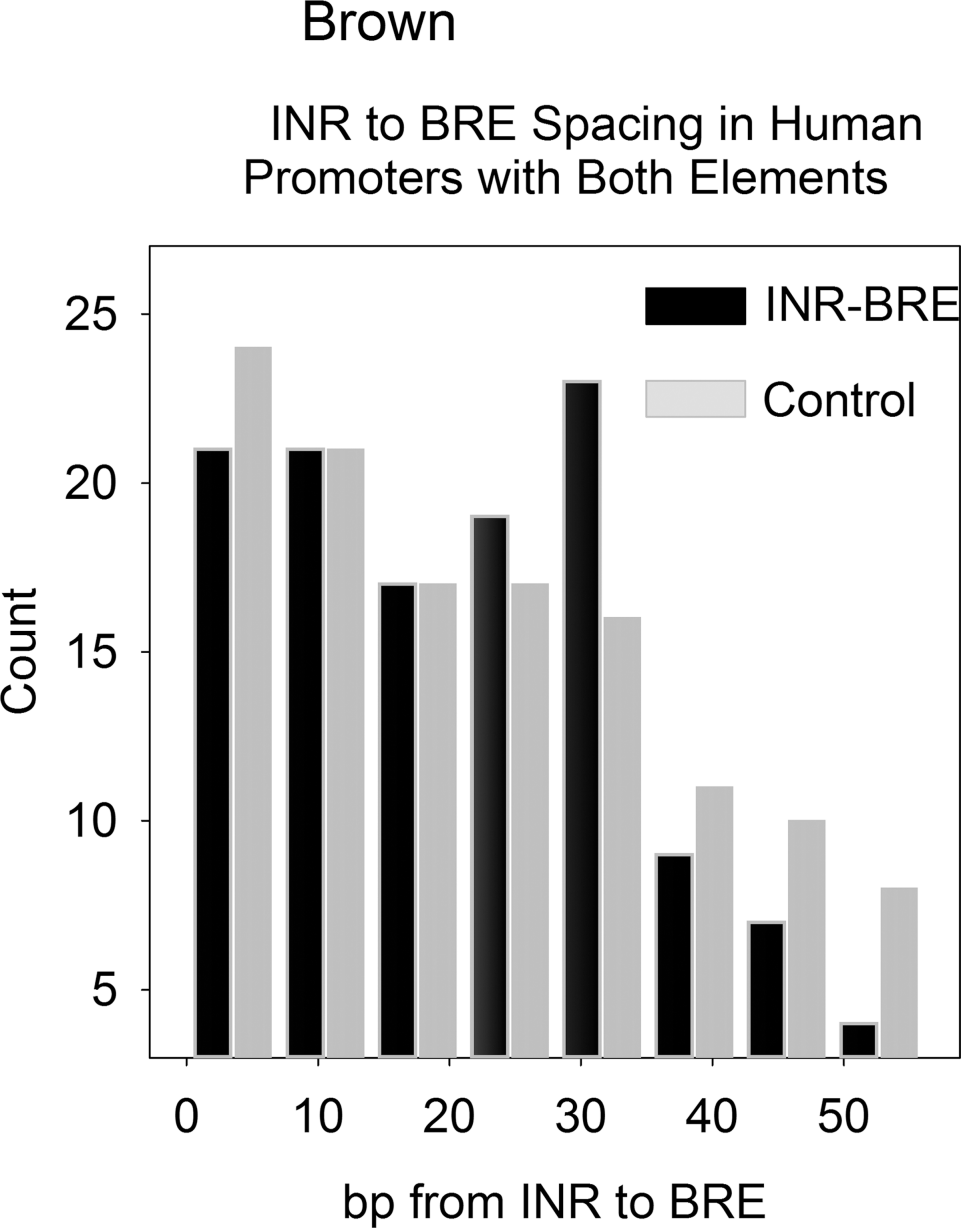
Histogram showing the INR-BRE spacing in the core promoter region (-60-+10bp) of the 121 database promoters where both elements are present (black bars). The control shows the results obtained when random distances were substituted for the determined ones in the same 70bp interval (gray bars). Note that preferred INR-BRE distances are in the range of 25-30bp with few distances above ~30bp.

Although the number of promoters with both INR and BRE elements is small, it was considered that analysis of this class might be revealing about how the two elements function together in initiation of transcription. The INR-BRE separation distance was therefore determined in the core region (-60-+10) of all promoters in the database that have both INR and BRE elements (121 promoters in all; 128 INR-BRE pairs). As a control, the spacings were compared with those between random numbers in the same 70bp interval. As expected, short spacings were more abundant than long ones in both INR-BRE and control distributions. The INR-BRE count exceeded the control only for separations of ~25-30bp (Fig 5). Other high abundance INR-BRE spacings occurred for all counts less than ~30bp suggesting the importance of this group of INR-BRE separations.

The distance between adjacent BRE elements was determined using the same strategy employed for INR-BRE. Beginning with all promoters in the database, those with more than one BRE in the core promoter were identified and the number of bp between adjacent BRE elements was plotted as a histogram. A total of 413 BRE-BRE pairs were examined. As a control, a similar plot was calculated using the separations between random numbers in a 70bp interval.

**Fig 6:**
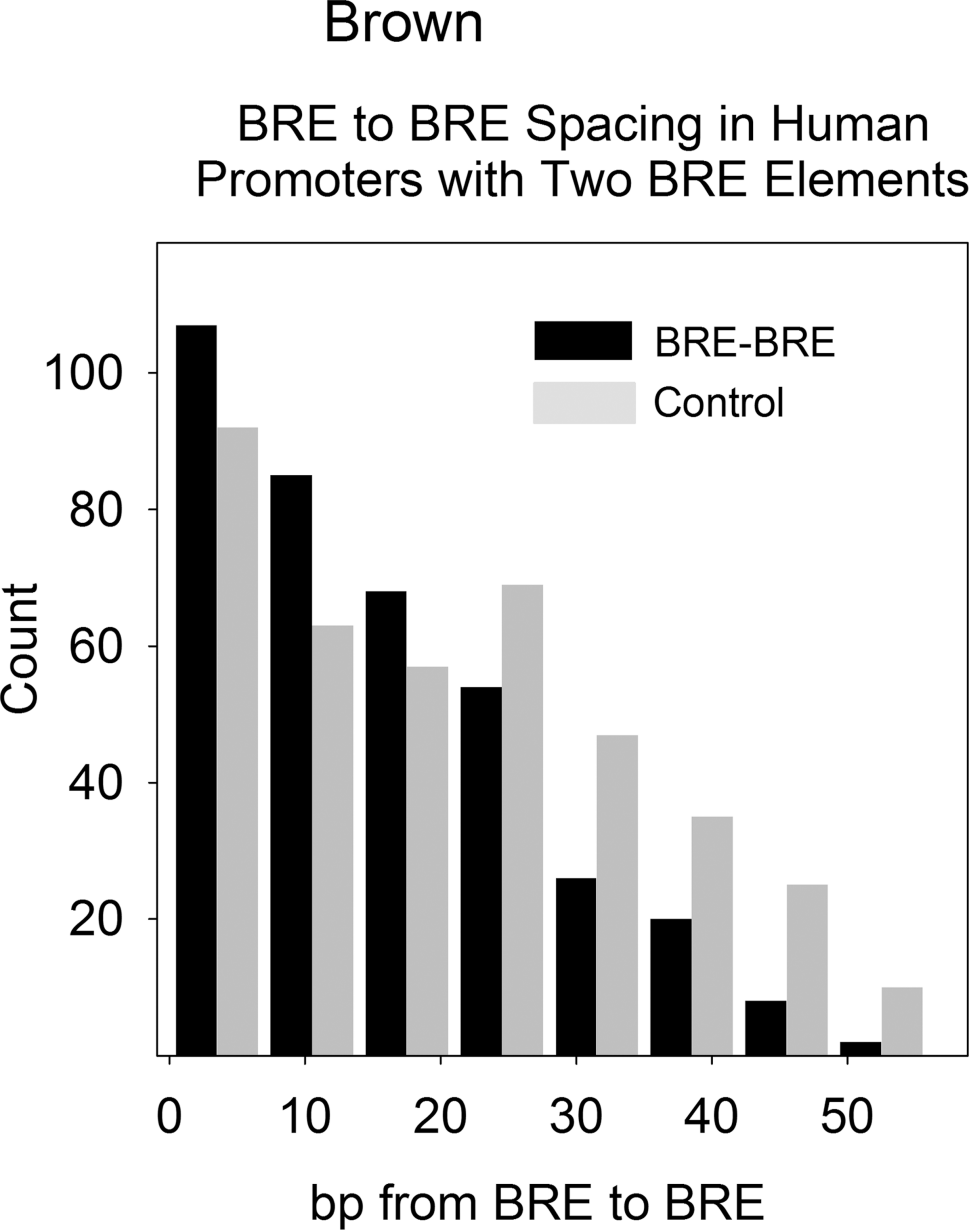
Histogram showing the BRE-BRE spacing in the core promoter region (-60-+10bp) of database promoters where more than one BRE element is present (black bars). The control shows the results obtained when random distances were substituted for the determined ones in the same 70bp interval (gray bars). Note that preferred BRE-BRE distances are in the range of ~1-15bp with few distances above ~40bp.

The results (Fig 6) showed that the count of BRE-BRE pair intervals was above background for spacings of less than ~15bp suggesting a tendency for adjacent BRE elements to be close together. Spacings of ~30bp or more were below the background in abundance. The results are interpreted to indicate that the protein factors that recognize BRE-BRE sequences, primarily TFIIB [23], function best when they are in close physical proximity to each other or function as a part of a group of BRE elements.

### Correlation of INR-BRE sequences with divergent transcription

Examination of the genes encoded by promoters containing both INR and BRE elements revealed an unexpected correlation with divergent transcription [24]. For this study, a gene was considered to be a protein coding gene or a non-coding transcript as long as expression of the transcript and its direction were annotated in the reference human genome (2013; hg38). Pseudogenes were not counted. Analysis included all 121 human genes found in the promoter database to have both INR and BRE elements in the core promoter. Genes transcribed in opposite directions were scored as positive in this analysis if the transcription start sites were within 2000bp of each other. Control experiments were performed with (1) genes randomly selected from the human genome; (2) genes whose promoter contained an INR element but no BRE; and (3) genes whose promoter contained a BRE element but no INR. Like the experimental gene set, control sets contained 121 genes (less any pseudogenes). Genes in the control sets were identified randomly with the number of genes from each chromosome matched to the number from the experimental set (i.e. the INR-BRE set).

The results showed 63 divergent and 58 non-divergent genes with INR-BRE promoters (Tables 2 and S1–S4). Lower counts and proportions were observed in the three control datasets (Table 2). Statistical analysis demonstrated that only in the INR-BRE promoters was the proportion of divergent genes significantly greater than that in the control samples. The observed correlation of INR-BRE promoters with divergent gene organization was not expected. It was anticipated that the two core elements would relate to the expression of the gene controlled and not its organization on the chromosome. The result suggests the proximity of INR and BRE elements may favor the creation of divergent transcription sites.

**Table 2:** Divergent transcription in genes with INR+BRE and control promoters.

	Gene Transcription				
Promoter Type	Divergent	Left or Right	Total	%Divergent	P value^a^
INR and BRE present	63	58	121	52%	<0.001
INR only	47	69	116	41%	0.121
BRE only	41	78	119	34%	0.543
Random sample^b^	35	86	114	29%	———

^a^ t test

^b^ All chromosomes sampled.

## Discussion

### Promoter database

The promoter database employed in this study was created with the goal of including the maximum number of promoters with emphasis on those driving annotated, functional genes. The prediction algorithm used, FirstEF [22], emphasizes promoters associated with CpG islands in proximity to the gene first exon [25, 26]. Emphasis on CpG islands favors capture of a high proportion of human promoters and this expectation was realized. The number of promoters identified, 13,406, was well above half of the known human genes. Emphasis on promoters within or near CpG islands has the consequence that TATA box-containing promoters are depleted as the TATA and CpG island promoter populations are thought to be weakly overlapping. It is expected therefore that TATA box genes are selectively depleted from the promoter population analyzed. Inclusion of promoters driving functional genes was favored in the database by selecting only FirstEF promoters near annotated, functional genes. Promoters were excluded if they were near pseudogenes or in chromosome regions lacking genes. Sequences downstream of the TSS were included in each downloaded promoter in case downstream INR and BRE elements might have an unanticipated function in control of gene expression.

The nature of the database employed here can be characterized by comparing it with other possibilities using the proportion of promoters found to have INR or BRE elements in the core. In the case of INR, the value obtained here, 9.2% (Table 1) is at the low end of the values reported previously (30%-49%; [27–29]). I suggest the difference may be due to the different sizes used for the core promoter region. The window used here, -20-+10 with respect to the TSS is much smaller than that used, for instance, by Yang et al. (-80-+80; [27]). In the case of BRE, the proportion reported here, 19.3% (Table 1) is in satisfactory agreement with the range (24%-25%) reported by Gershenzon and Ioshikhes ([29]).

### Locations of INR and BRE elements

A regular feature of the results reported here is the very different distribution of INR promoter elements in individual chromosomes. Chromosome dependence was also observed in the case of BRE distributions (Figs 1 and 2). Three different distributions were observed for INR and two for BRE. The distributions are sufficiently distinct that they are likely to reflect a fundamental feature of chromosome evolution or function. Possibilities include (1) the unique natural history of individual chromosomes; (2) a chromosome-specific structural feature; and (3) the different locations of each chromosome within the nucleus [30–32]. No systematic effects on the character of the encoded genes were noted on chromosomes that differed in INR or BRE element distribution.

The character of all three INR distributions was unexpected. The V distribution was reported for all human INR promoter elements [33], but the flat and sloped distributions have not been reported previously. All three INR distributions seem to be inconsistent with a role for INR at or very near the TSS. The V distribution in particular shows a minimum of INR abundance right at the TSS where its proposed function suggests it would be a maximum. Similarly, the flat and sloped distributions show no maximum or other singular feature at the TSS. Three potential explanations suggest themselves for the above observations: (1) INR could function over a wider core promoter range than is usually considered. This would be the case, for example, if factors other than the INR sequence itself would affect the location where INR could function. (2) It may be that INR elements promote a high level of transcription initiation while lower levels are more appropriate for most genes. A lower level of transcription might not require involvement of an INR element. (3) There may be other as yet unrecognized elements that can substitute for INR to promote transcription. No single explanation for the observed INR distributions seems to me to stand out at the present time.

The distributions observed for BRE elements more closely resemble the results expected for a core promoter component. In a majority of chromosomes (16 of 22) there is a maximum in the distribution of BRE elements at or near the core promoter (central peak distribution; see Fig 2 and S2 Powerpoint). Such a distribution is expected of an element that functions within the core promoter. Another explanation must apply in the case of the six chromosomes with skewed BRE promoter distributions. BRE elements located remotely from the core promoter may have a function that can be expressed outside the core region or they may not function in control of gene expression at all.

### INR and BRE elements in the core promoter

As in the case of the whole promoter study, chromosome dependence was also observed when analysis of INR and BRE locations were focused on the core promoter region only. Flat and bimodal distributions were observed with INR while flat plus trimodal and multimodal were observed with BRE. In both the INR and BRE cases, flat was the predominant distribution observed among the 22 chromosomes able to be classified. The flat distribution indicates that the abundance of INR or BRE elements among the promoters examined does not vary markedly with location within the core promoter. This finding is in disagreement with results from in vitro studies with single promoters where INR is found to be most active when it is located at the TSS and where BRE is located 35-39bp upstream [23, 34]. I suggest the different results may be due to the greater number of promoters examined in the present study. The flat distributions observed here further suggest that both INR and BRE elements are able to function at varying distances from the TSS.

Perhaps the most interesting feature of the core compared to the entire promoter analyses is the existence of clusters of INR and BRE elements upstream of the TSS (see Figs 3 and 4, and also S3 and S4 Powerpoints). Two such clusters of INR elements are found in the case of six chromosomes; in all six the spacing between INR clusters is ~10-20bp. Three BRE clusters are observed in six chromosomes with a spacing of ~20bp. Up to six BRE clusters were detected in four chromosomes where the cluster spacing is ~10bp. The existence of distinct clusters suggests elements in a cluster may have something in common. Possibilities include a common structural feature or perhaps a relationship among the genes driven by elements in the same cluster.

The fact that clusters differ in their distance to the TSS indicates that distinct mechanisms must be used to decode them. Binding in the BRE-TSS interval by differing sets of transcription factors suggests itself as the underlying mechanism. The periodicity observed in some cases (e.g. the multimodal BRE distributions) raises the possibility that a repeating unit of transcription factors may be involved.

### Promoters with both INR and BRE elements

Although the number of promoters with both INR and BRE elements is very small, it was considered attractive to examine them as such rare combinations might have unique properties. In fact, the observed number of INR+BRE promoters was lower than the abundance expected by chance. The statistical expectation (i.e. the measured INR promoter frequency (9.2%) X the BRE frequency (19.3%) is 1.8%. In contrast, the measured value (0.9%) is ~2-fold lower suggesting that INR+BRE-containing promoters are depleted overall compared to the chance expectation. The presence of an INR or a BRE in a promoter may therefore suppress the presence of the other. The observed spacing between INR and BRE elements in INR+BRE promoters (~25-30bp; see Fig 5) indicates that the two elements do not need to be in close physical proximity to exert any effects the pair may have.

It was unexpected to detect a correlation of INR+BRE-containing core promoters with divergently-transcribed genes (Table 2; [24, 35–39]). The result suggests the novel possibility that core promoter elements may function in genome organization as well as in control of gene expression. The observations in Table 2 indicate further that the correlation with divergent transcription applies to core promoters containing both INR and BRE elements. No similar correlation was observed with core promoters containing only INR or BRE.

### Promoters with two or more BRE elements

In contrast to the abundance of core promoters with both INR and BRE elements, the abundance of cores with two BRE elements is in agreement with the statistical expectation. 413 were found (Fig 6) while 500 were expected by chance (i.e. 2592 core promoters with one BRE X 0.193, the proportion of core promoters containing a BRE). This result indicates that the presence of a BRE element in the core promoter does not have a marked effect on whether a second one is present. The measured spacing between adjacent BRE elements (~1-15bp; see Fig 6) supports the view that BRE elements can function in close proximity to each other.

## Conclusions

The goal of the present study was to determine whether focusing on a large number of human promoters would reveal novel features about transcription initiation. Using a database of more than 13,000 promoters, analysis was restricted to the INR and BRE elements of the core promoter. The results demonstrated a chromosome-specific pattern to the way INR elements are distributed within promoter sequences. Chromosome specificity was also observed in the distribution of promoter BRE sequences. The results suggest that characteristic INR and BRE distributions are due to the distinct natural history of individual chromosomes, to a chromosome-specific structural feature or to the different locations of each chromosome within the nucleus [30–32].

A related study was performed to examine the location of INR and BRE elements within the core promoter. The results showed that a large proportion of core promoters lack an INR or a BRE element entirely. ~90% lack an INR and ~80% lack a BRE. The findings therefore support the view that all or most human promoters have the non-canonical rather than the canonical character and lack an INR or BRE element in the core promoter. In most promoters, some other feature must perform the function(s) INR and BRE perform in promoters that have them.

Finally, I performed an analysis of the small number of core promoters that have both an INR element and a BRE (121 promoters compared to >13,000 in the database). A correlation was found relating such promoters with divergent transcription in the adjacent genes. More than 50% of INR+BRE promoters were found to be located in a position where they could drive divergently-transcribed gene pairs. The result raises the possibility, for the first time, that core promoter elements can have a function in chromosome organization as well as in initiation of transcription.

## Supporting information

S1 Powerpoint Location of INR elements in database promoters of individual human chromosomes. (PPTX)

S2 Powerpoint Location of BRE elements in database promoters of individual human chromosomes. (PPTX)

S3 Powerpoint Location of INR elements in database core promoters of individual human chromosomes. (PPTX)

S4 Powerpoint Location of BRE elements in database core promoters of individual human chromosomes. (PPTX)

S1 Table Divergent transcription in human promoters containing both INR and BRE elements. (DOCX)

S2 Table Divergent transcription in human promoters containing INR but not BRE elements. (DOCX)

S3 Table Divergent transcription in human promoters containing BRE but not INR elements. (DOCX)

S4 Table Divergent transcription in randomly selected human gene promoters. (DOCX)

## Abbreviations

RNAPII: DNA-dependent RNA polymerase II
TSS: transcription start site
PIC: pre-initiation complex
TF: transcription factor
