# Supplementary material for: Control of human gene expression: High abundance of divergent transcription in genes containing both INR and BRE elements in the core promoter
Source: PLoS One. 2018 Aug 23;13(8):e0202927. doi: 10.1371/journal.pone.0202927 (PMC6107252; doi:10.1371/journal.pone.0202927)

## Slide 1
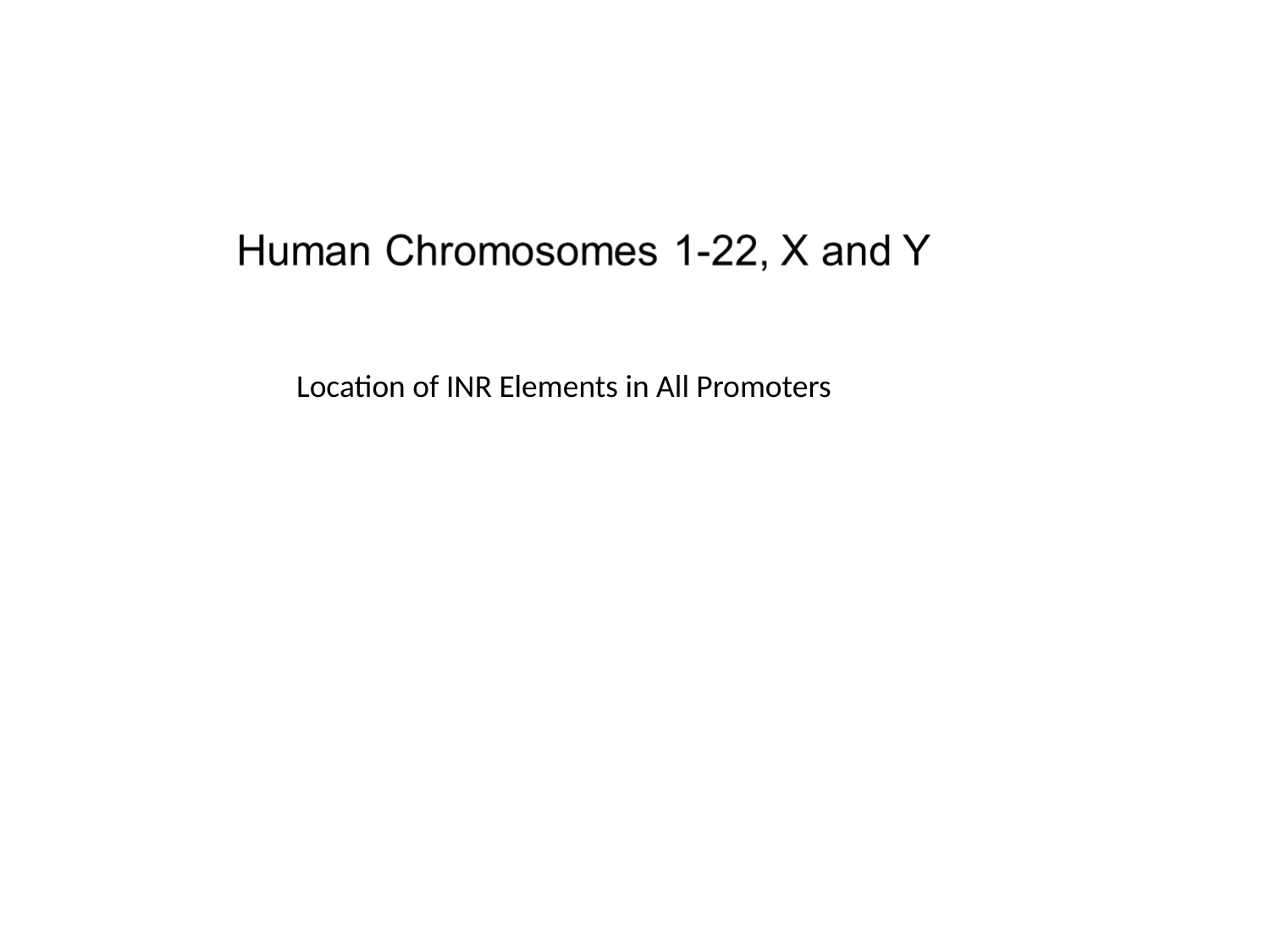

Location of INR Elements in All Promoters

## Slide 2
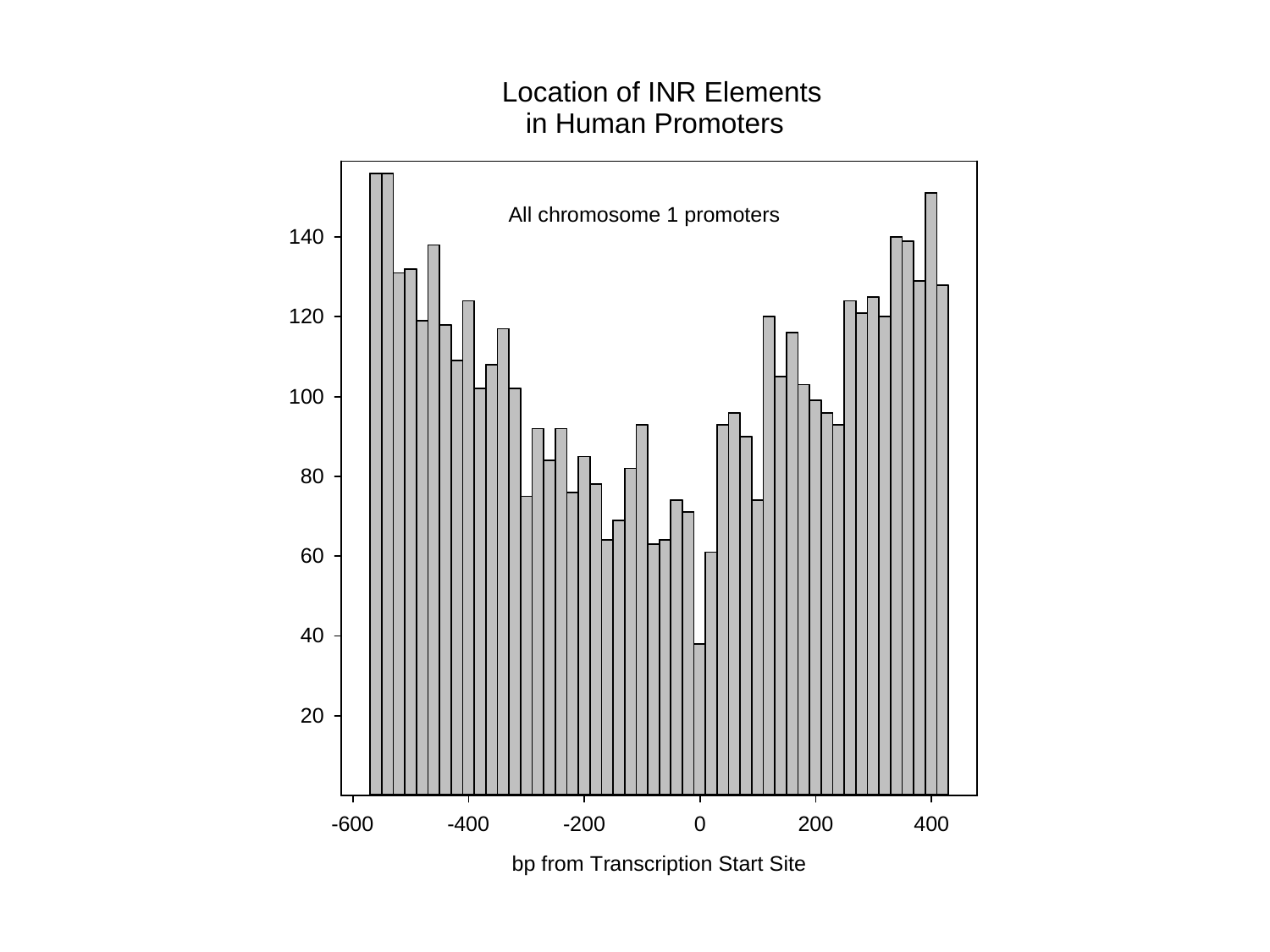

## Slide 3
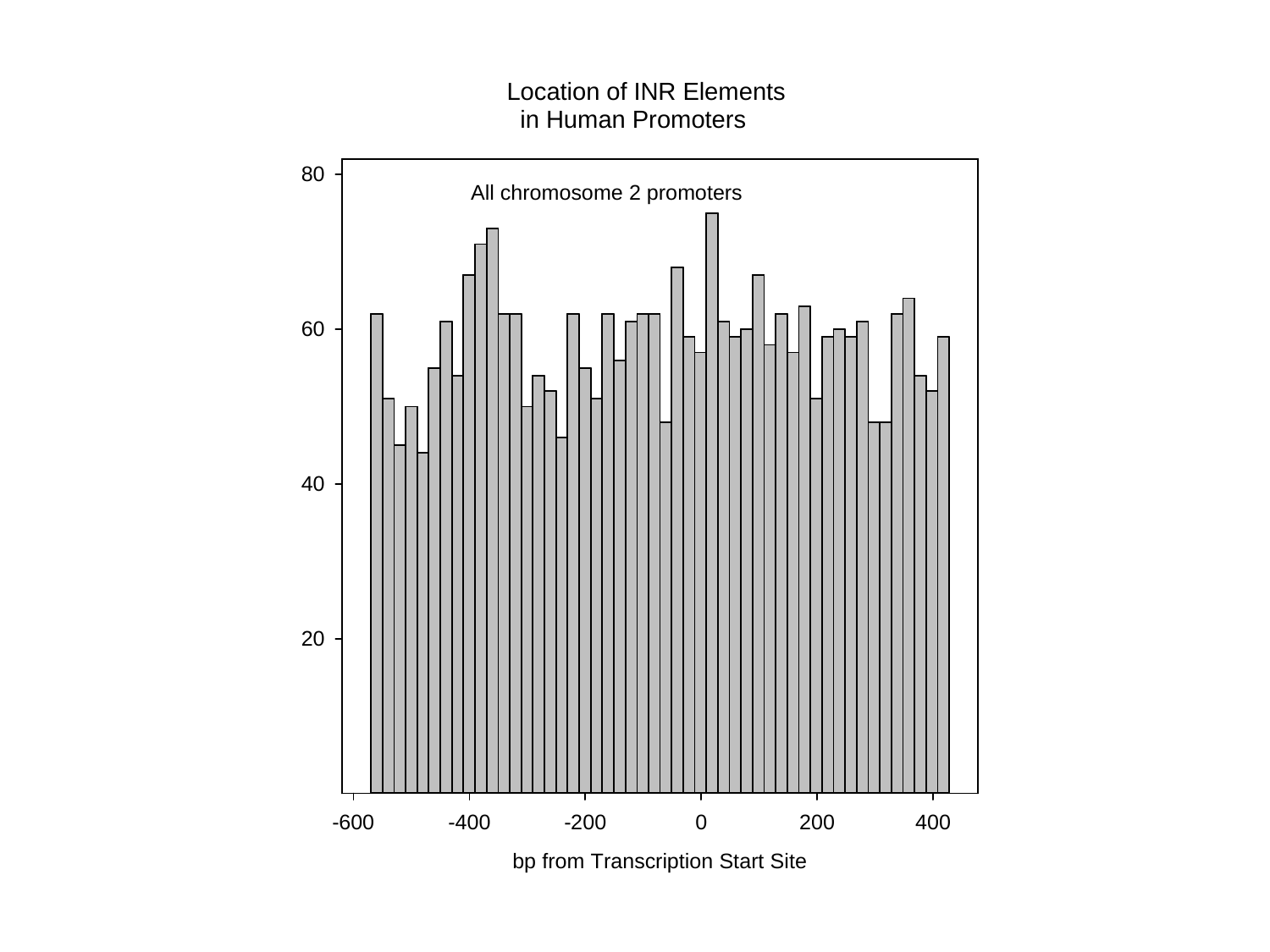

## Slide 4
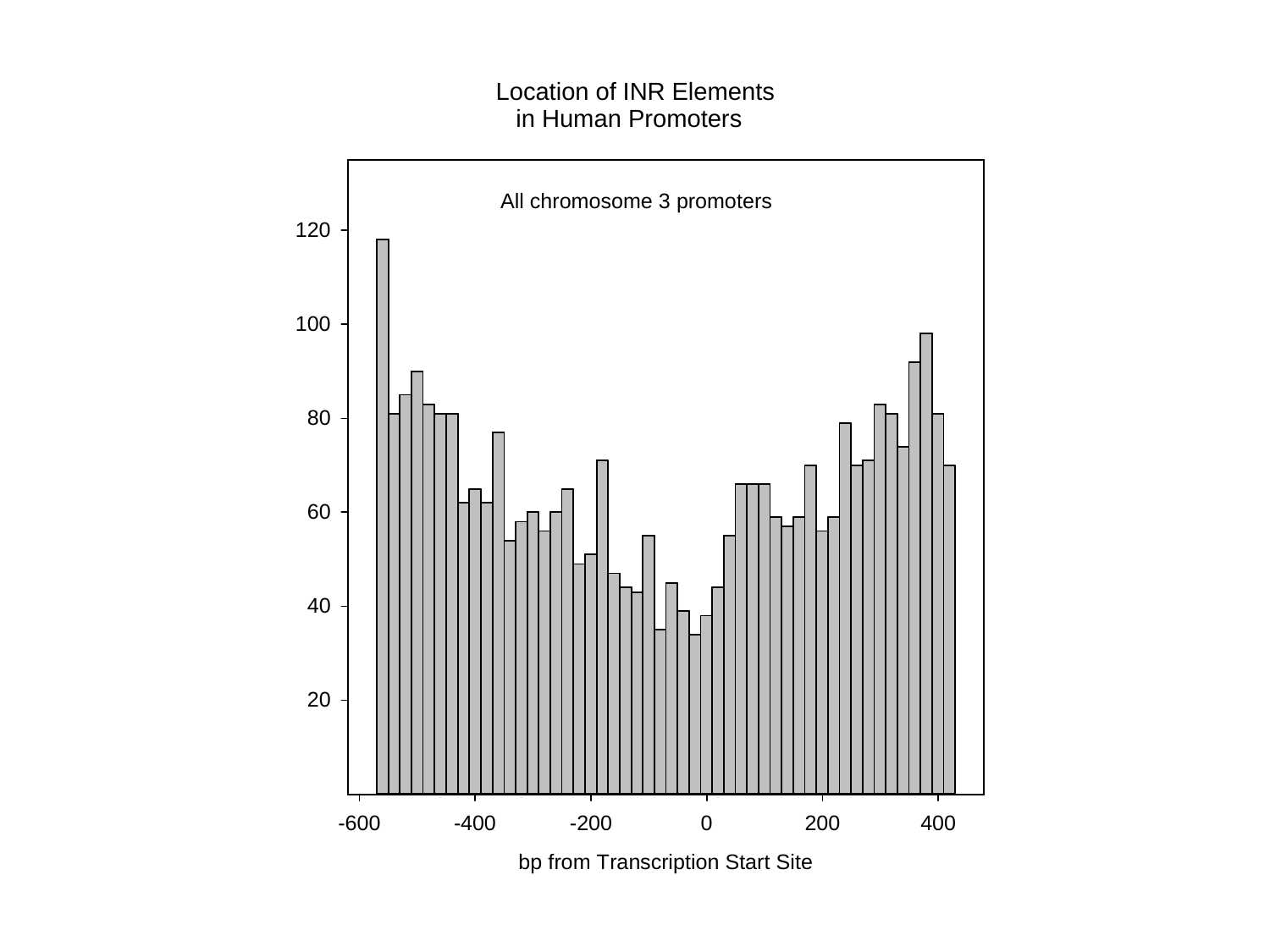

## Slide 5
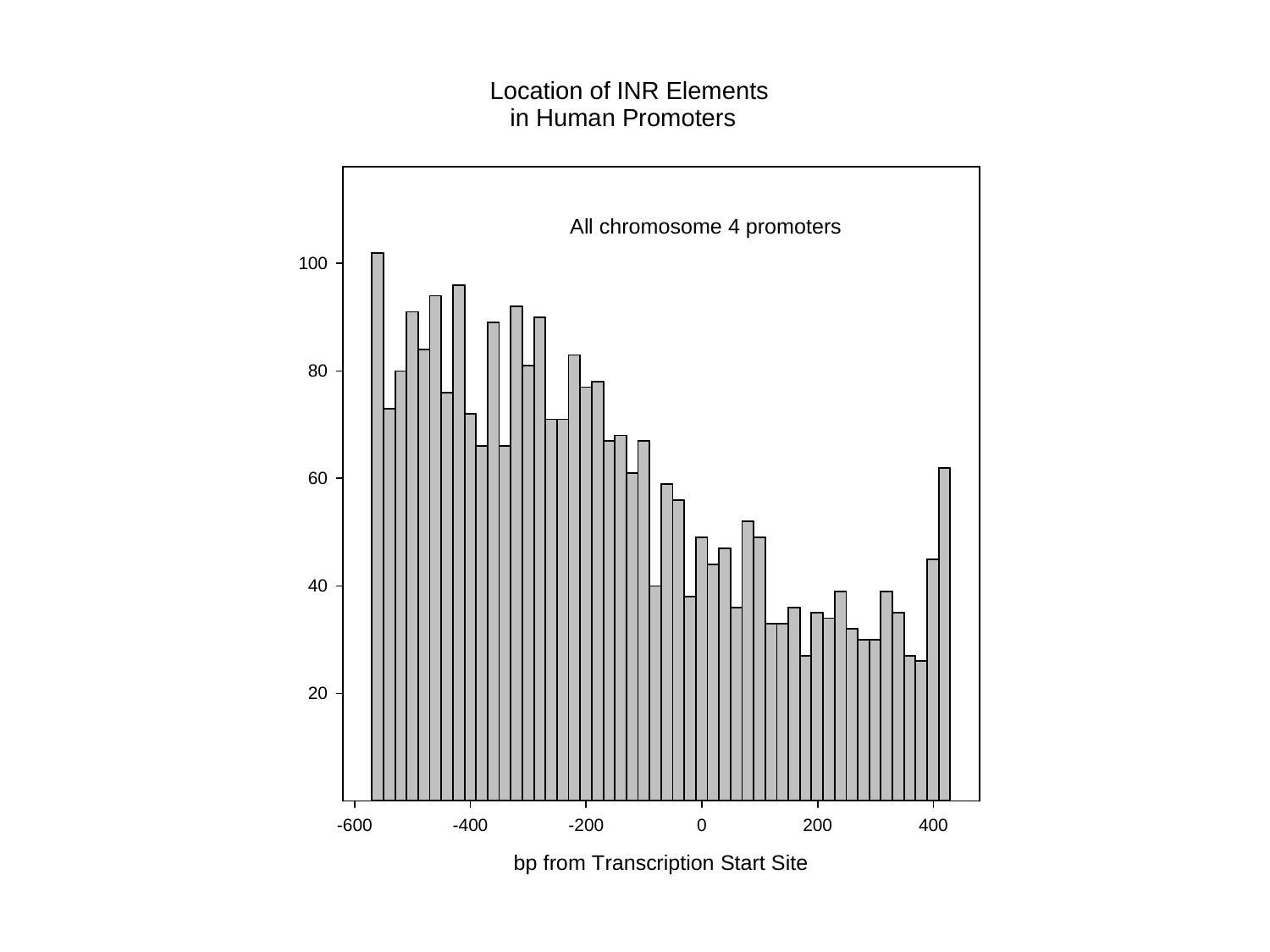

## Slide 6
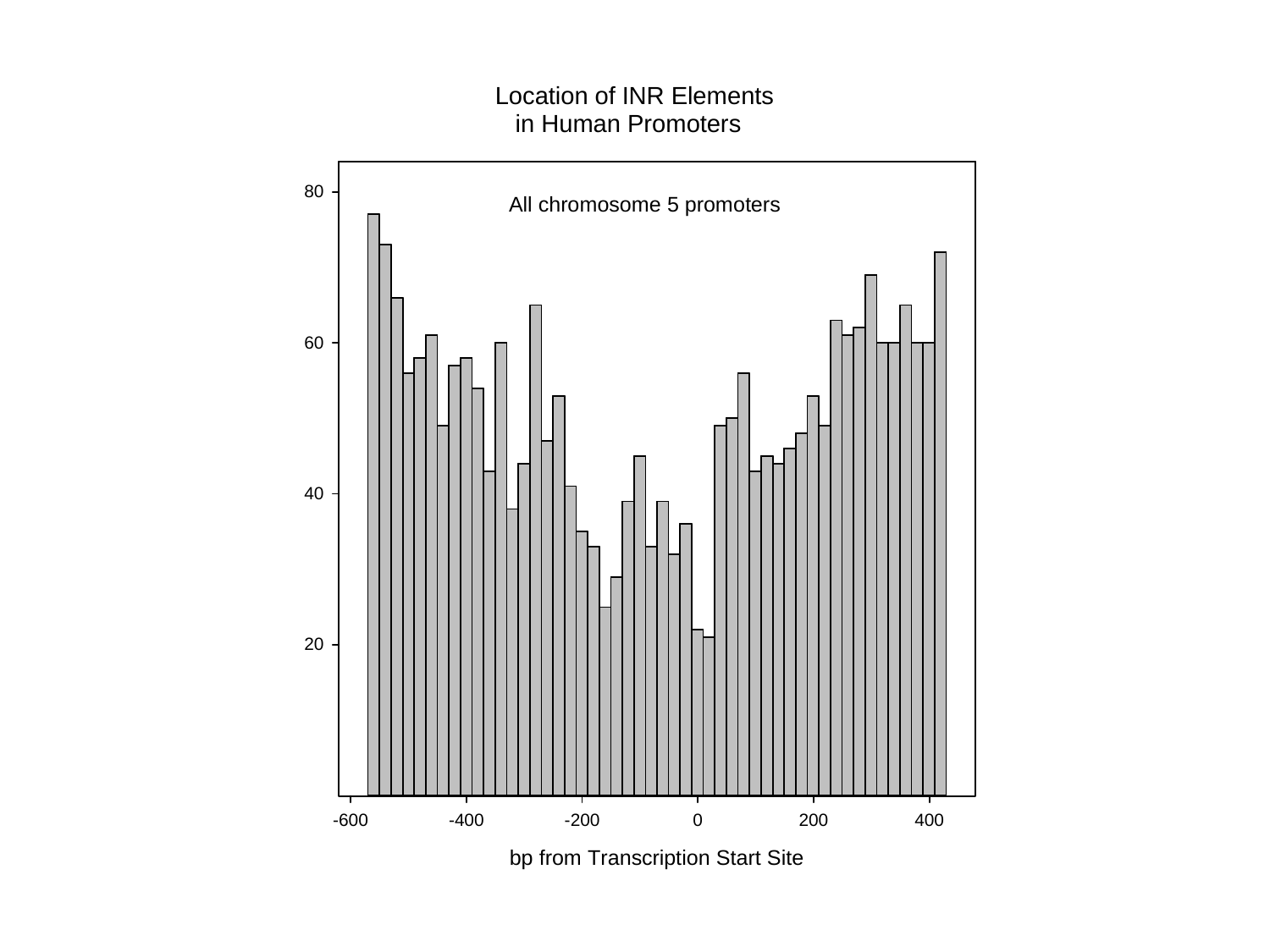

## Slide 7
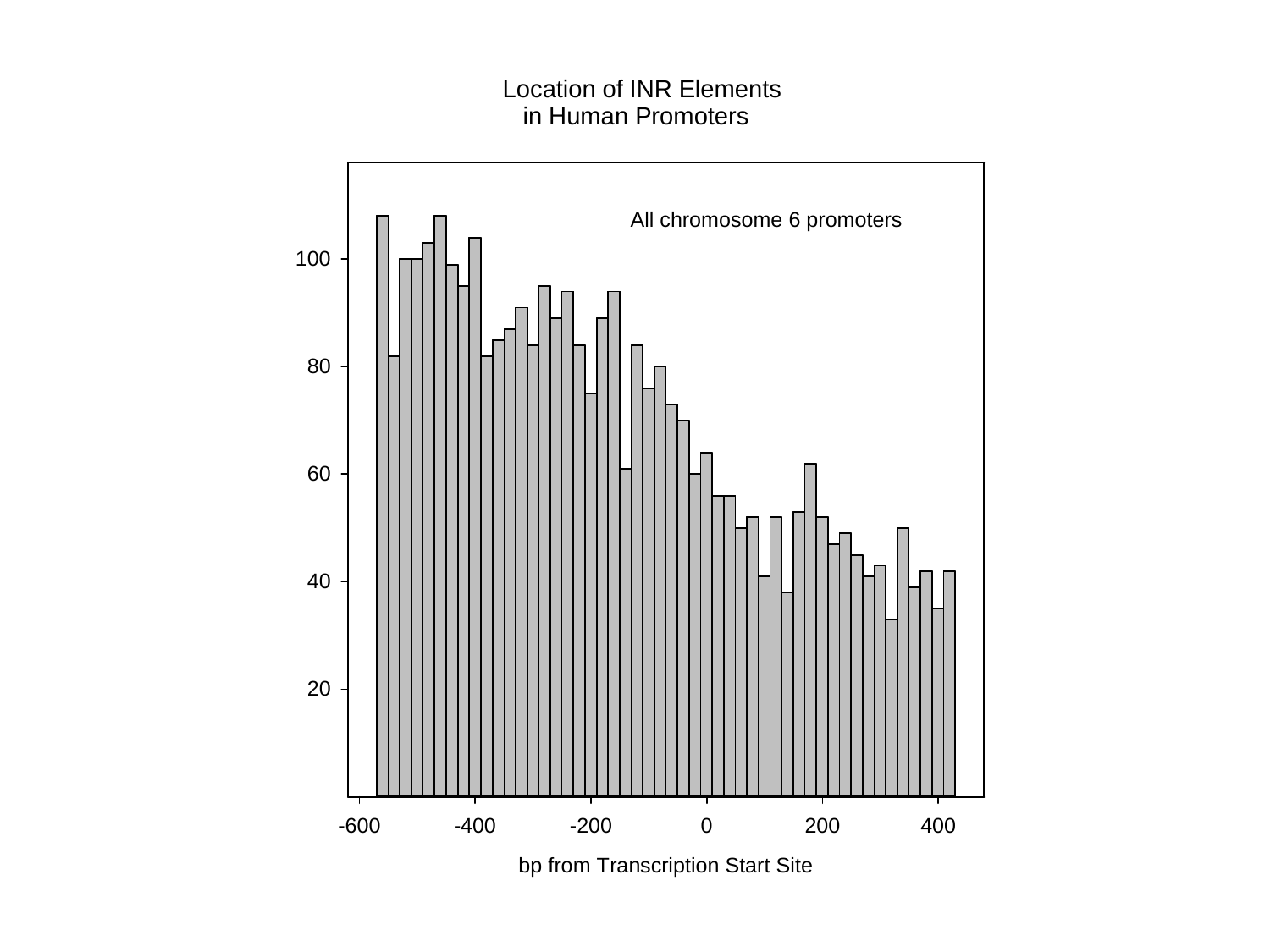

## Slide 8
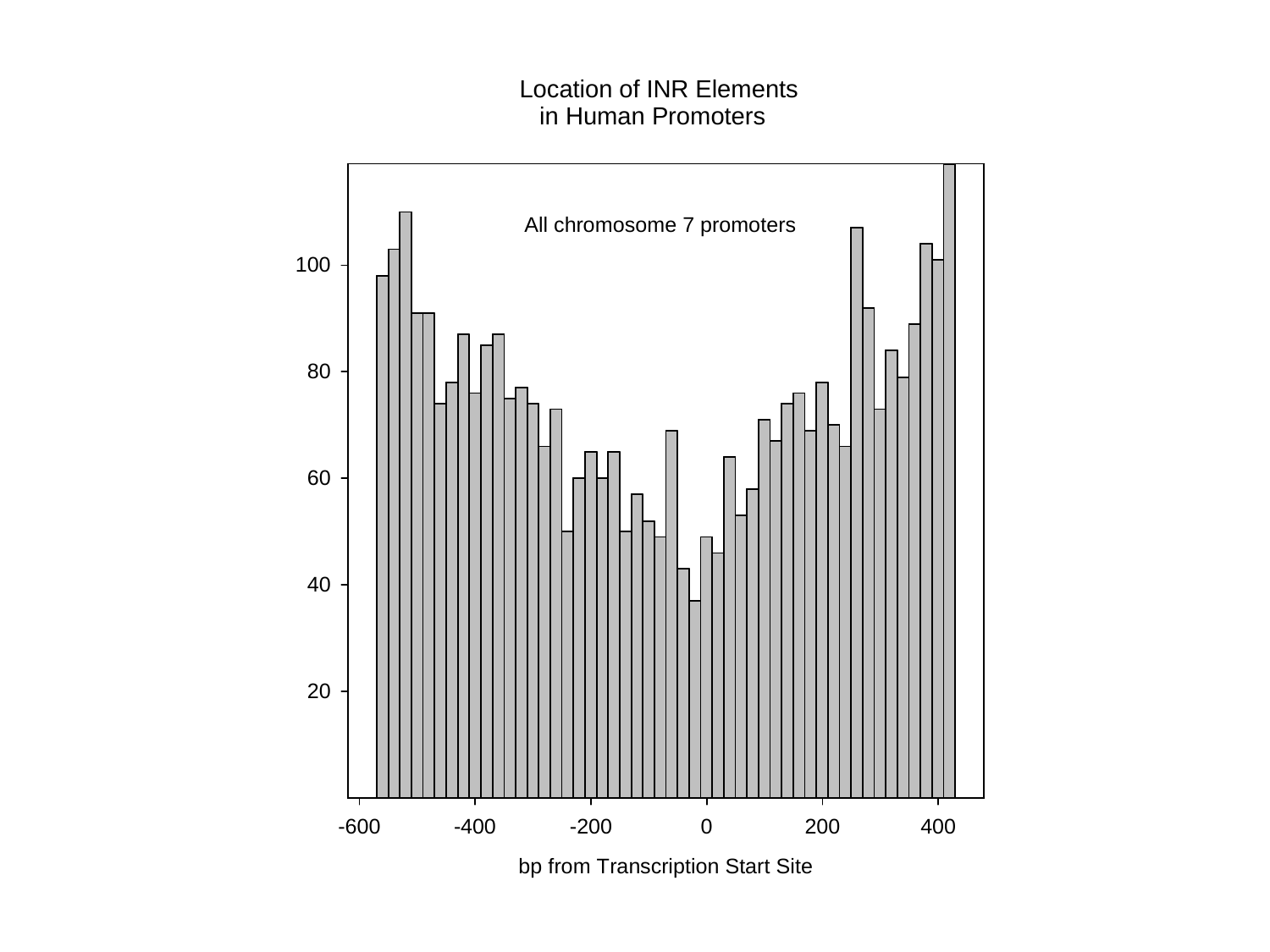

## Slide 9
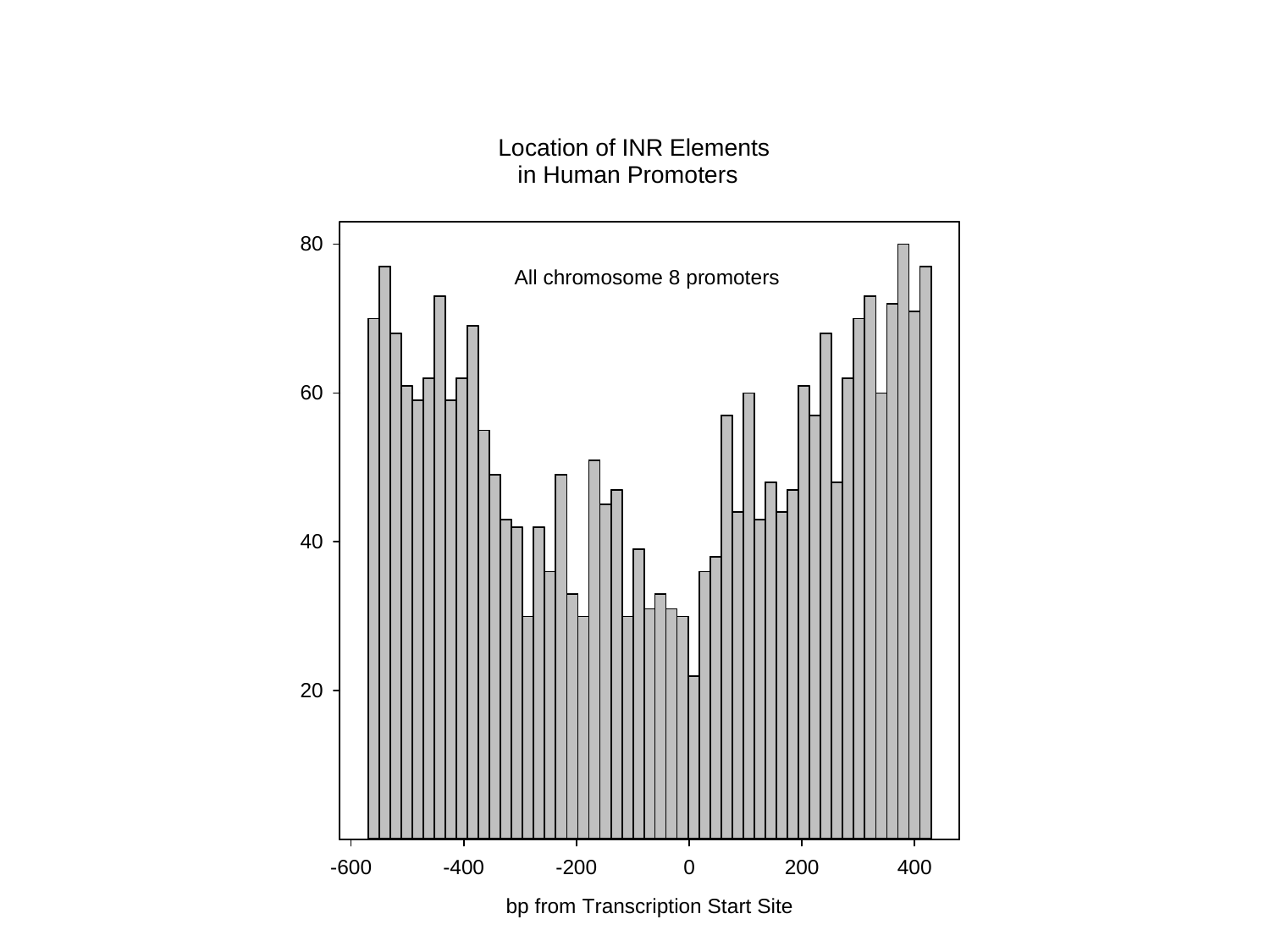

## Slide 10
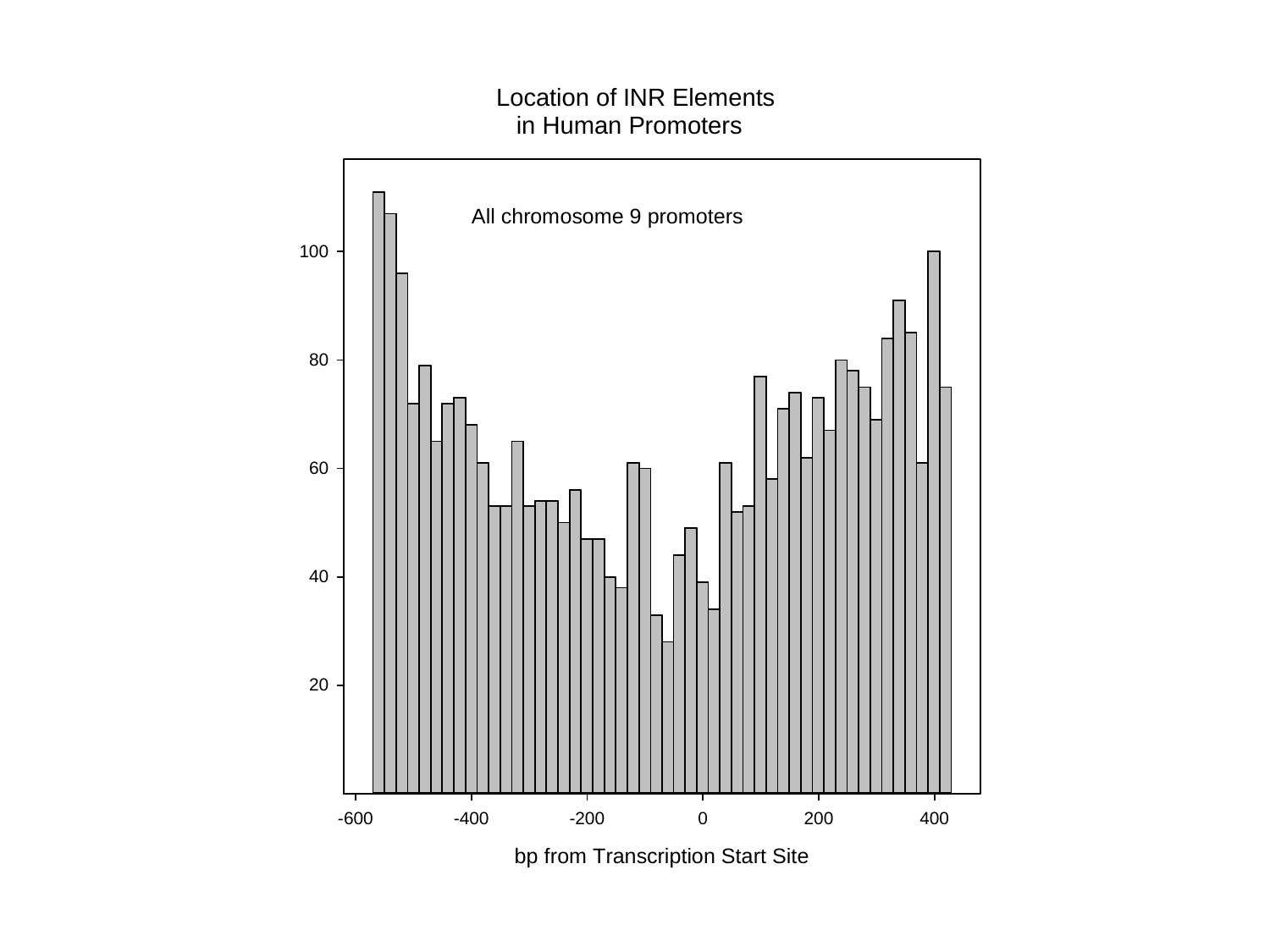

## Slide 11
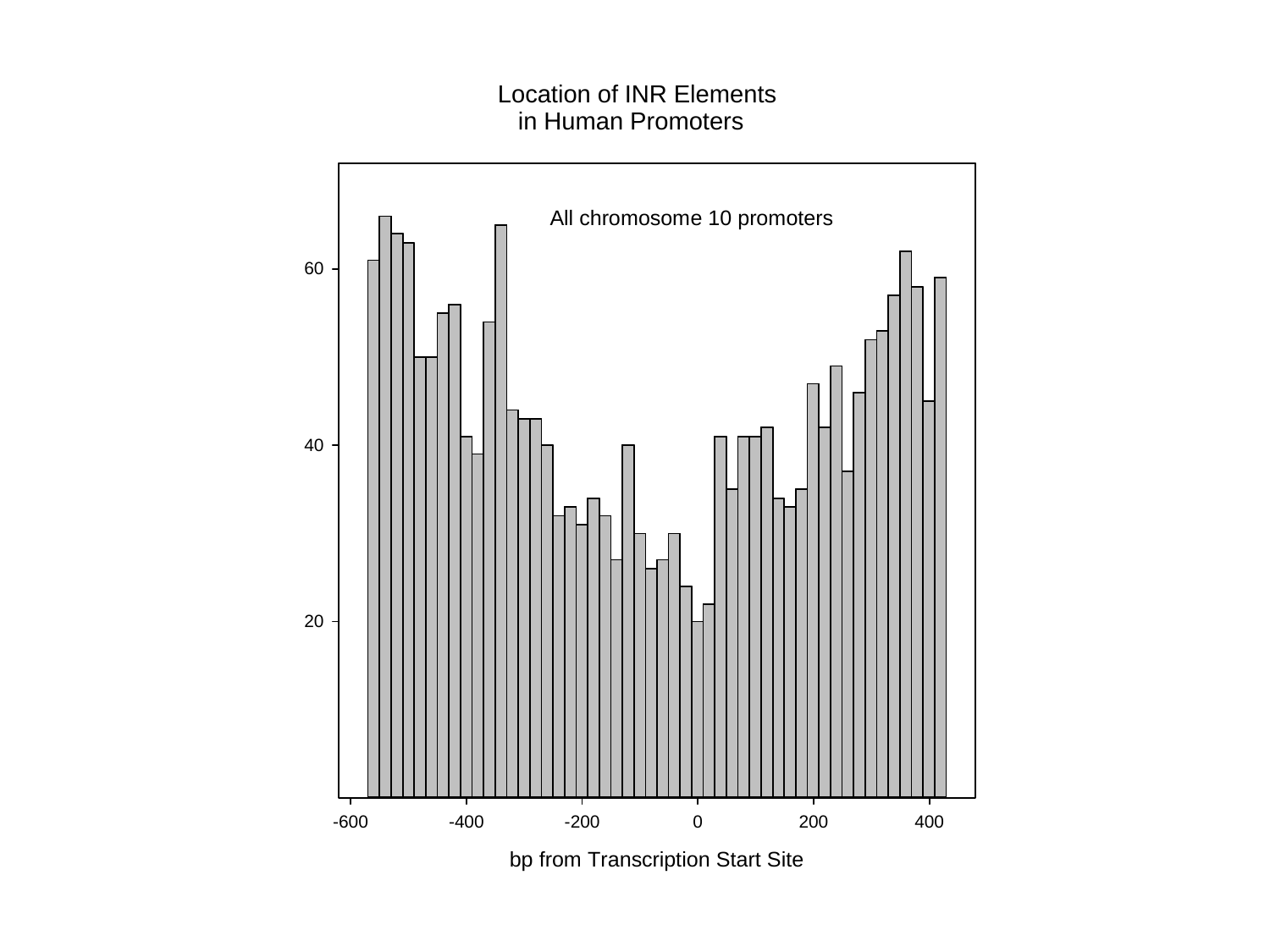

## Slide 12
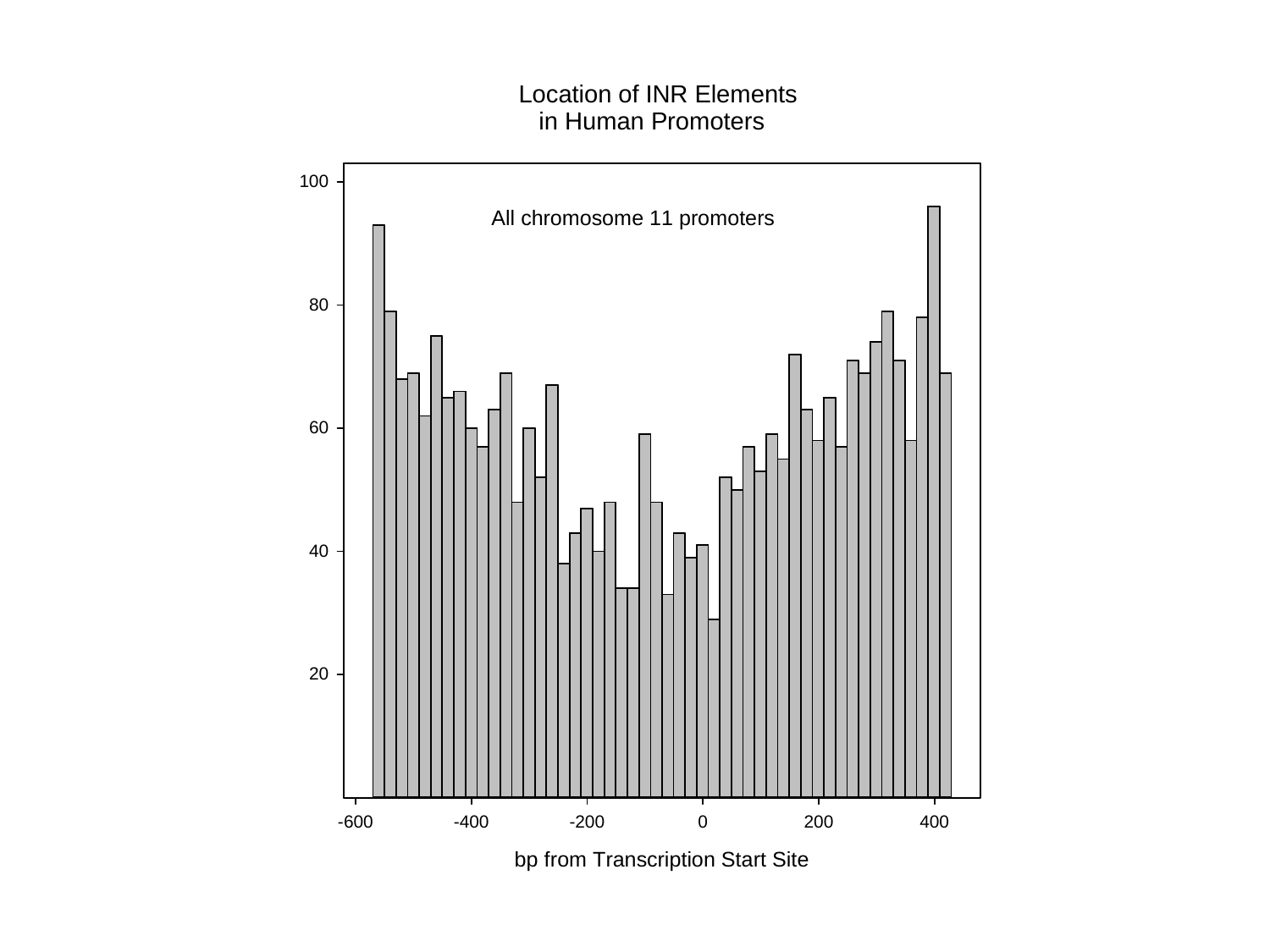

## Slide 13
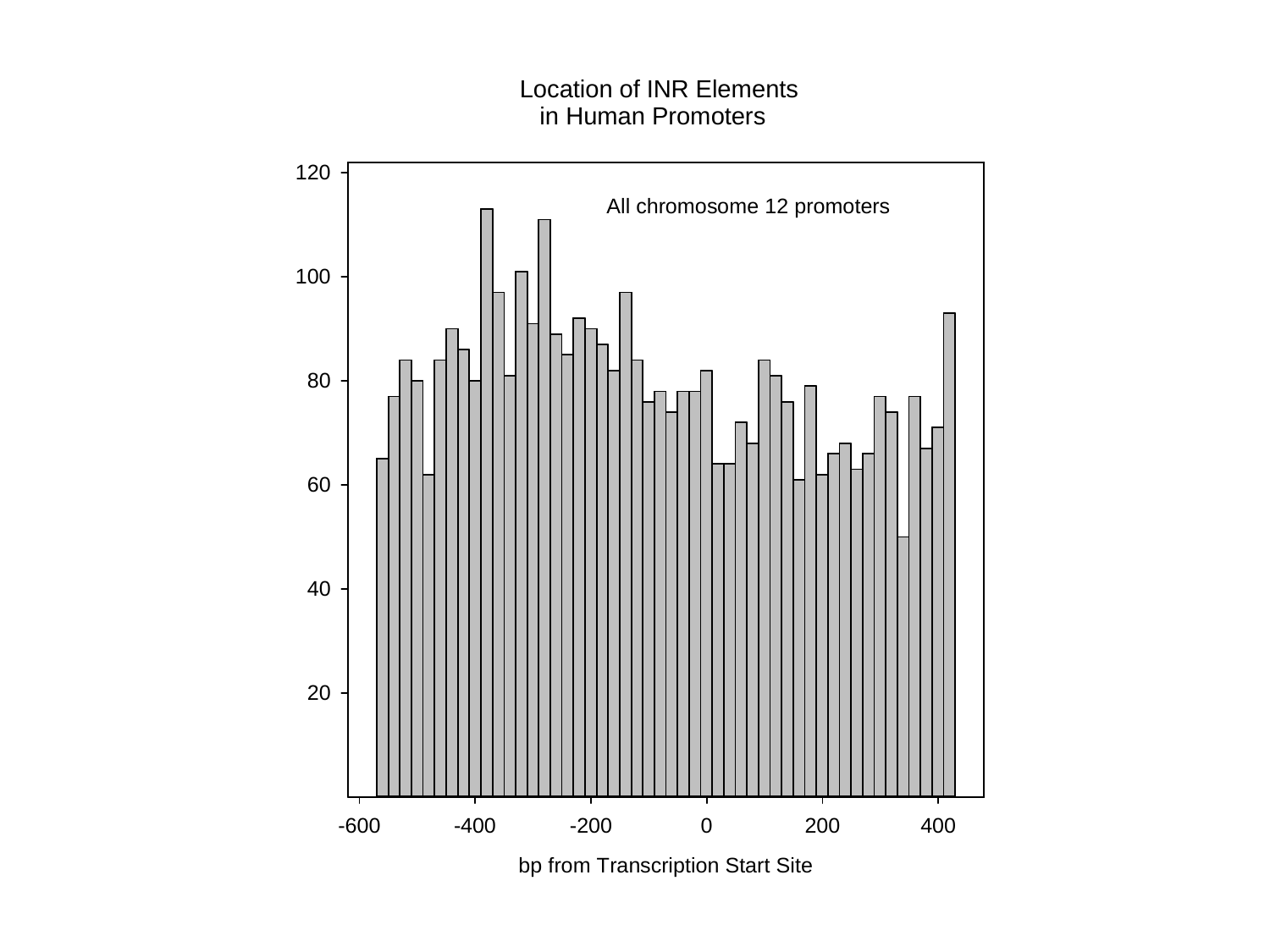

## Slide 14
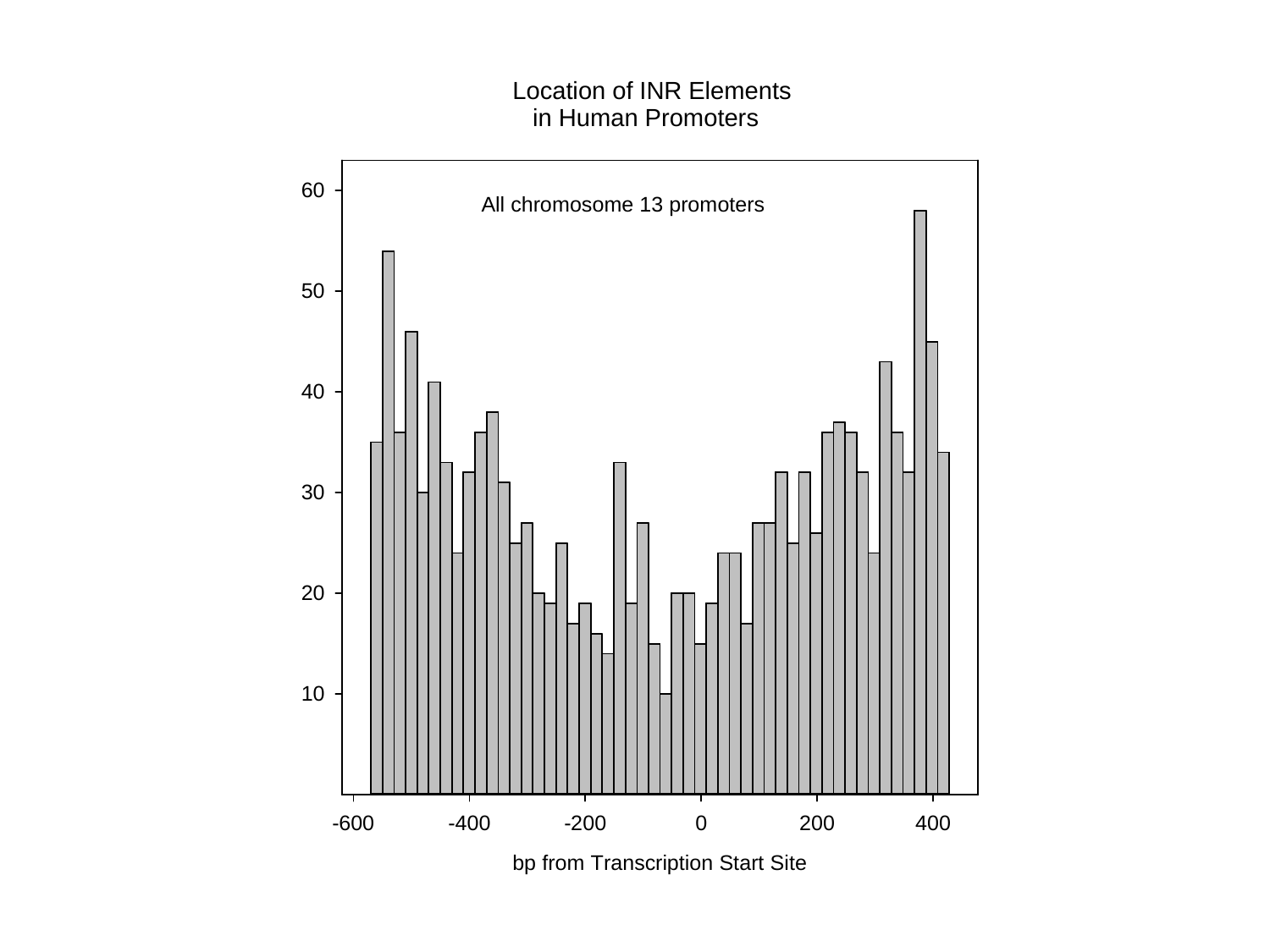

## Slide 15
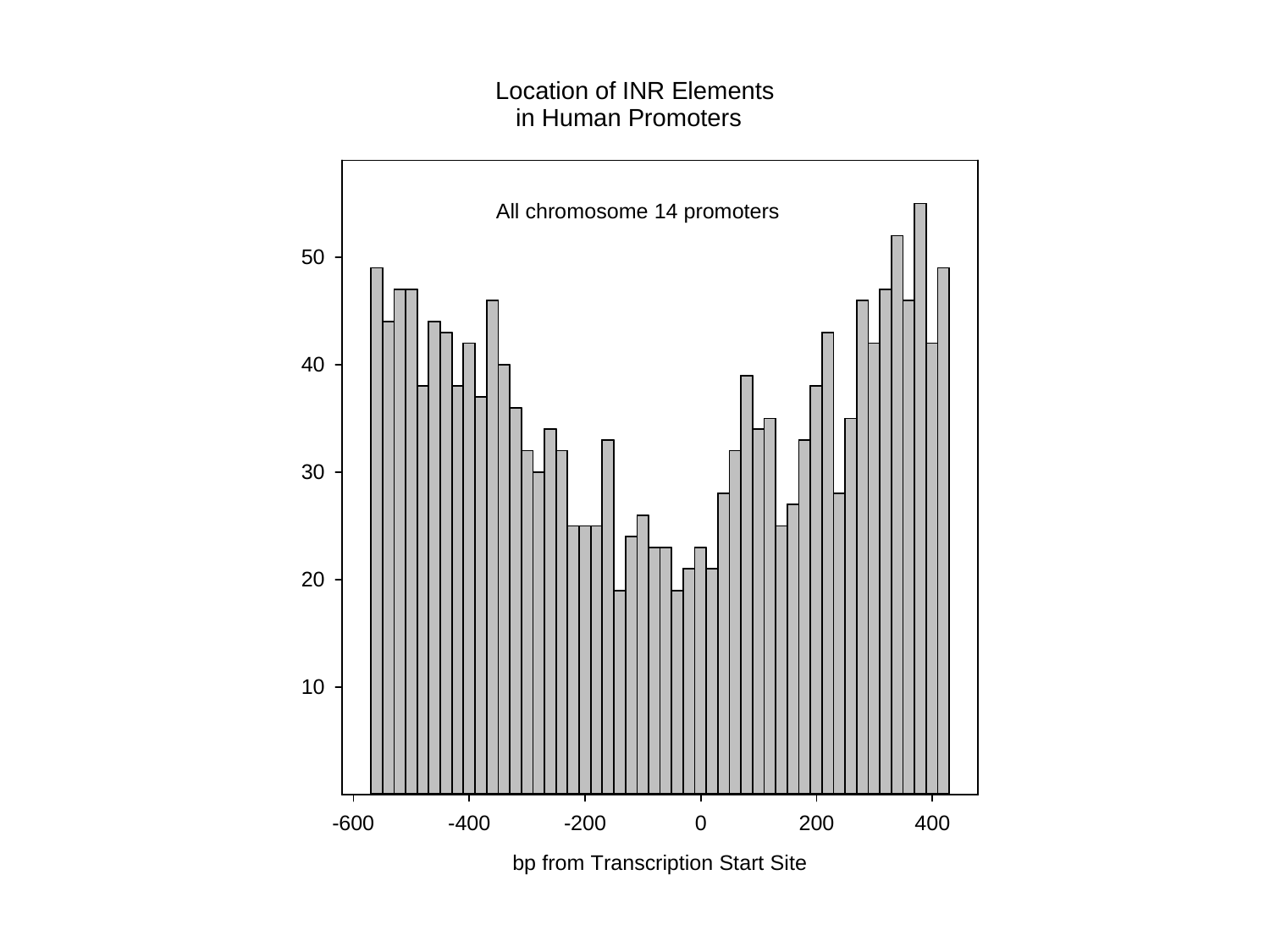

## Slide 16
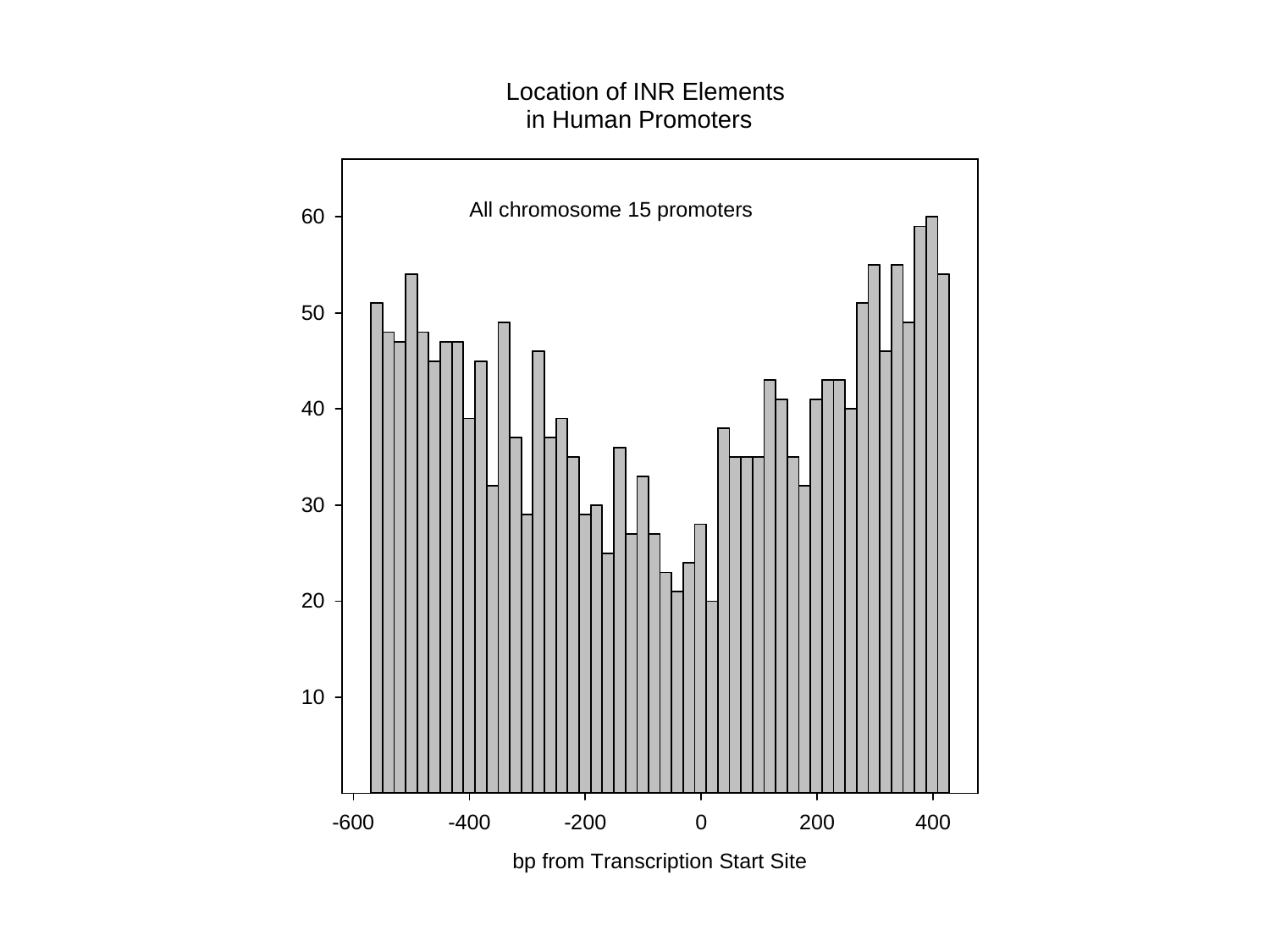

## Slide 17
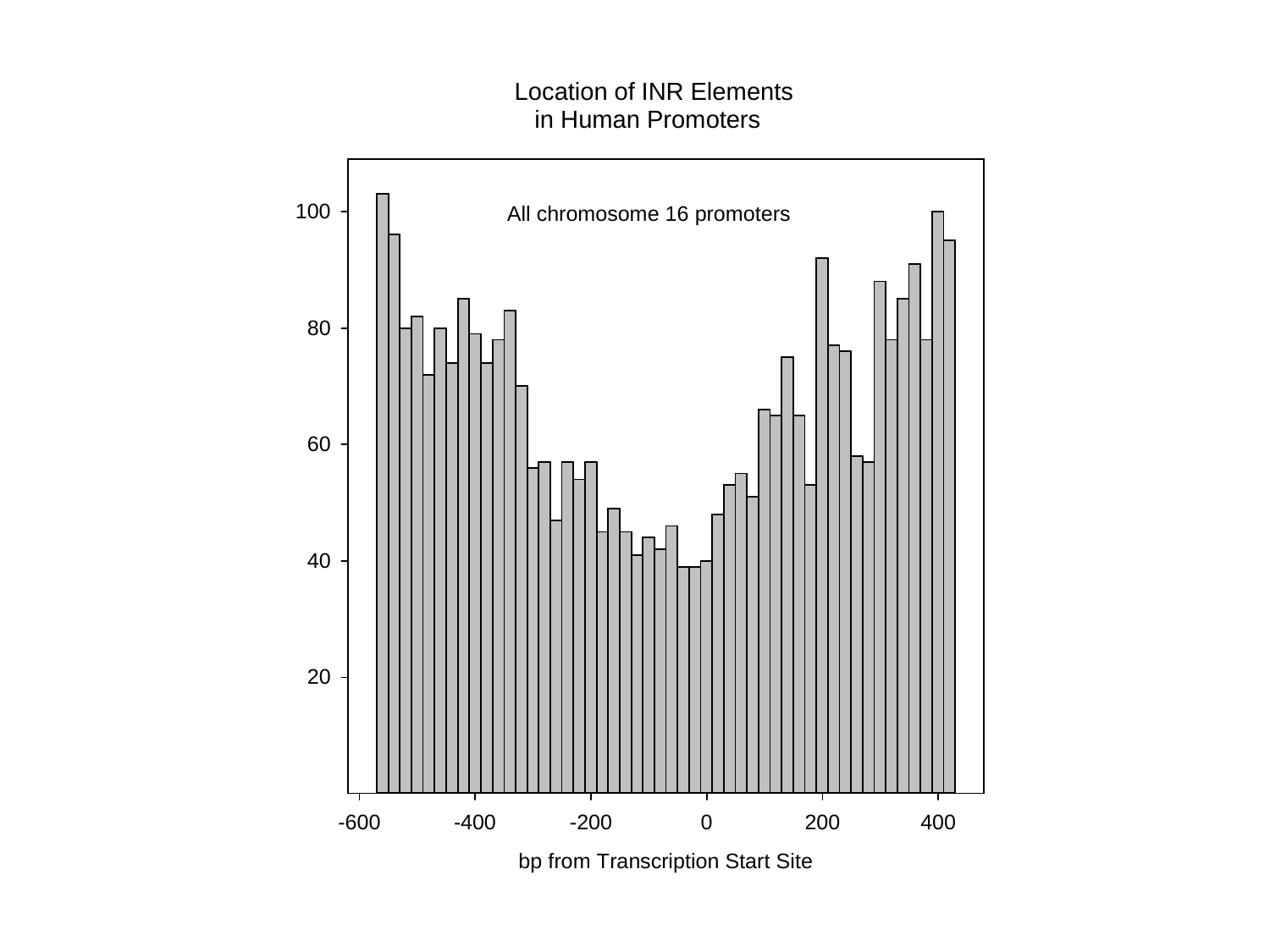

## Slide 18
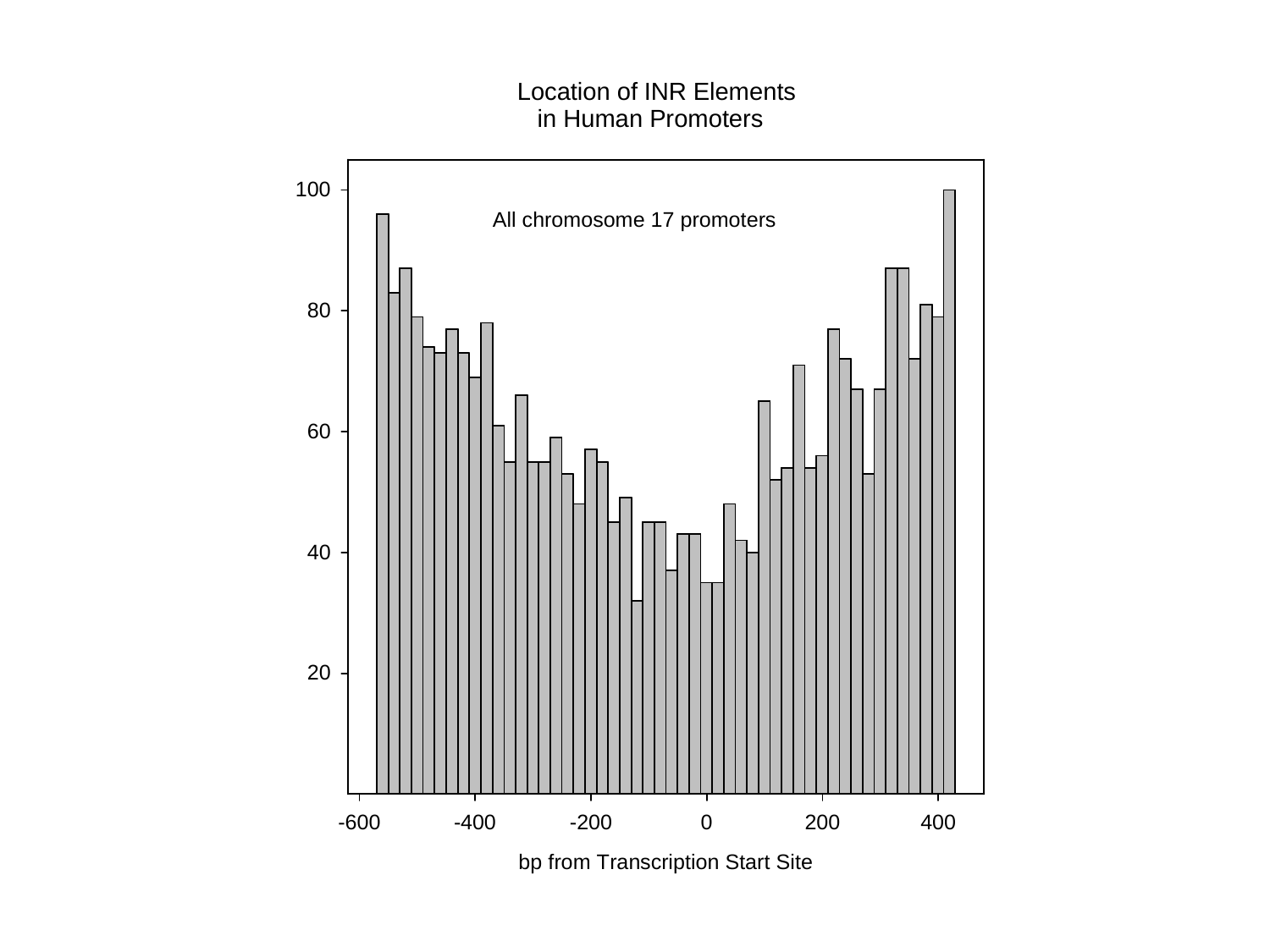

## Slide 19
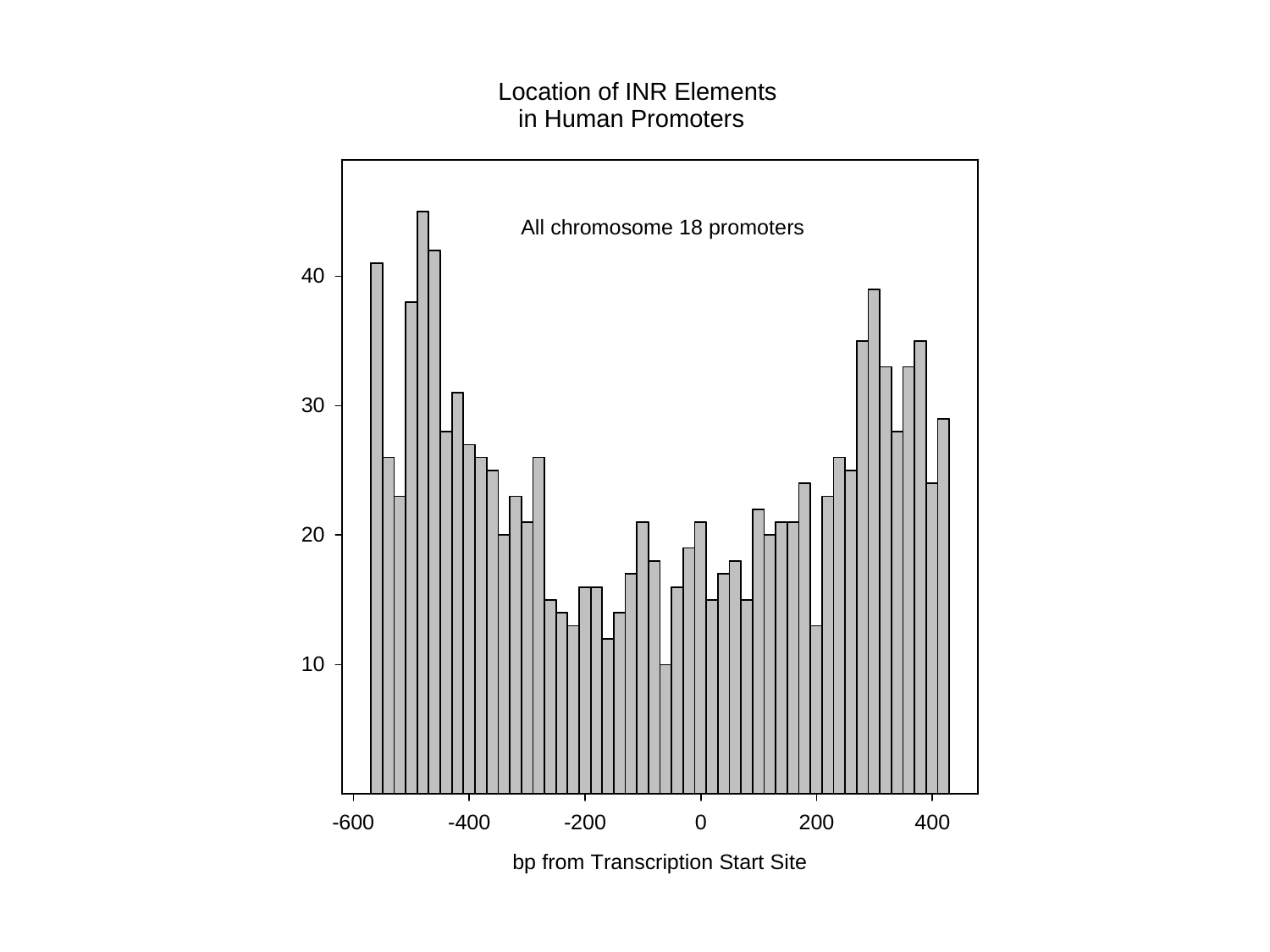

## Slide 20
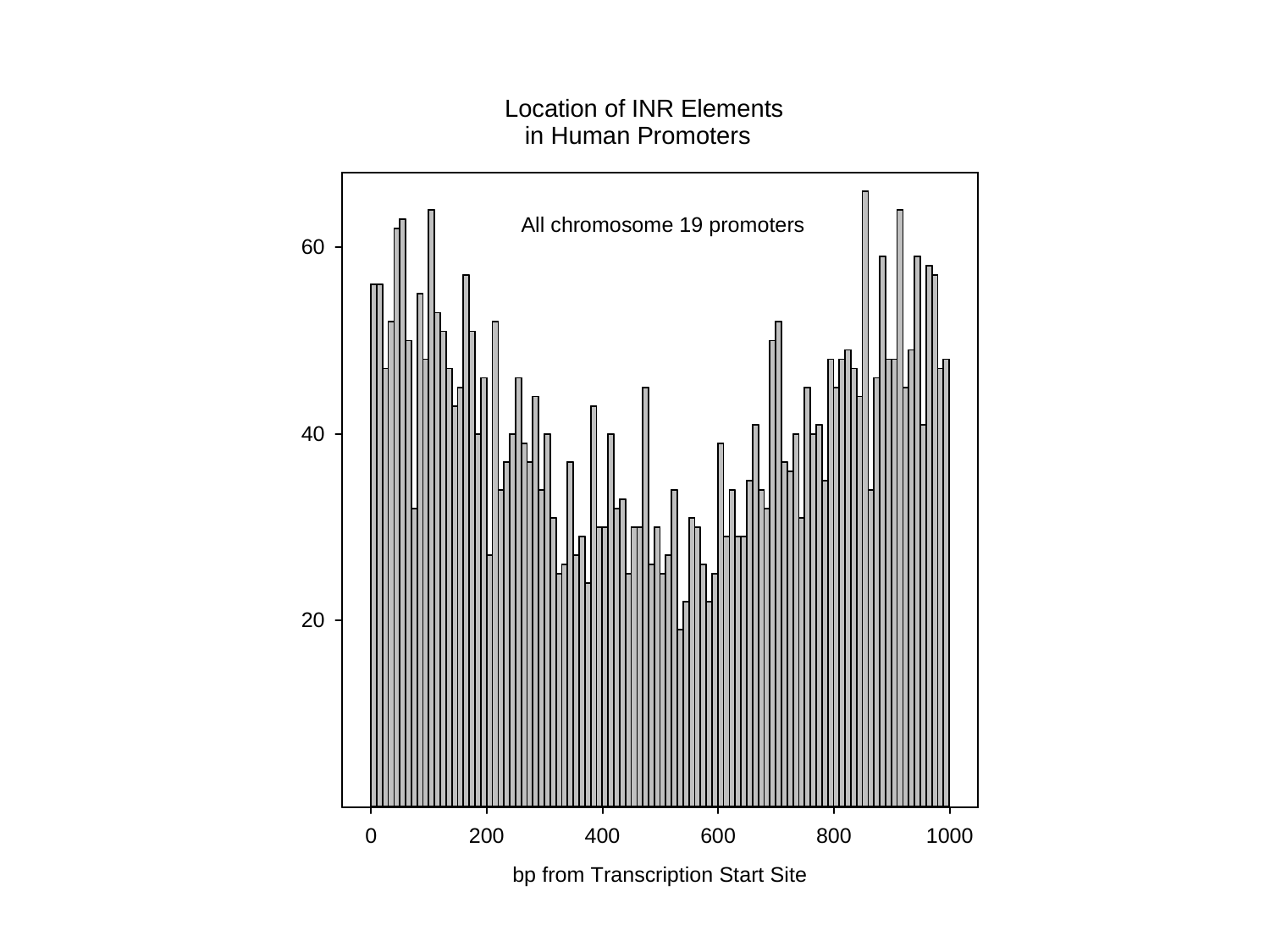

## Slide 21
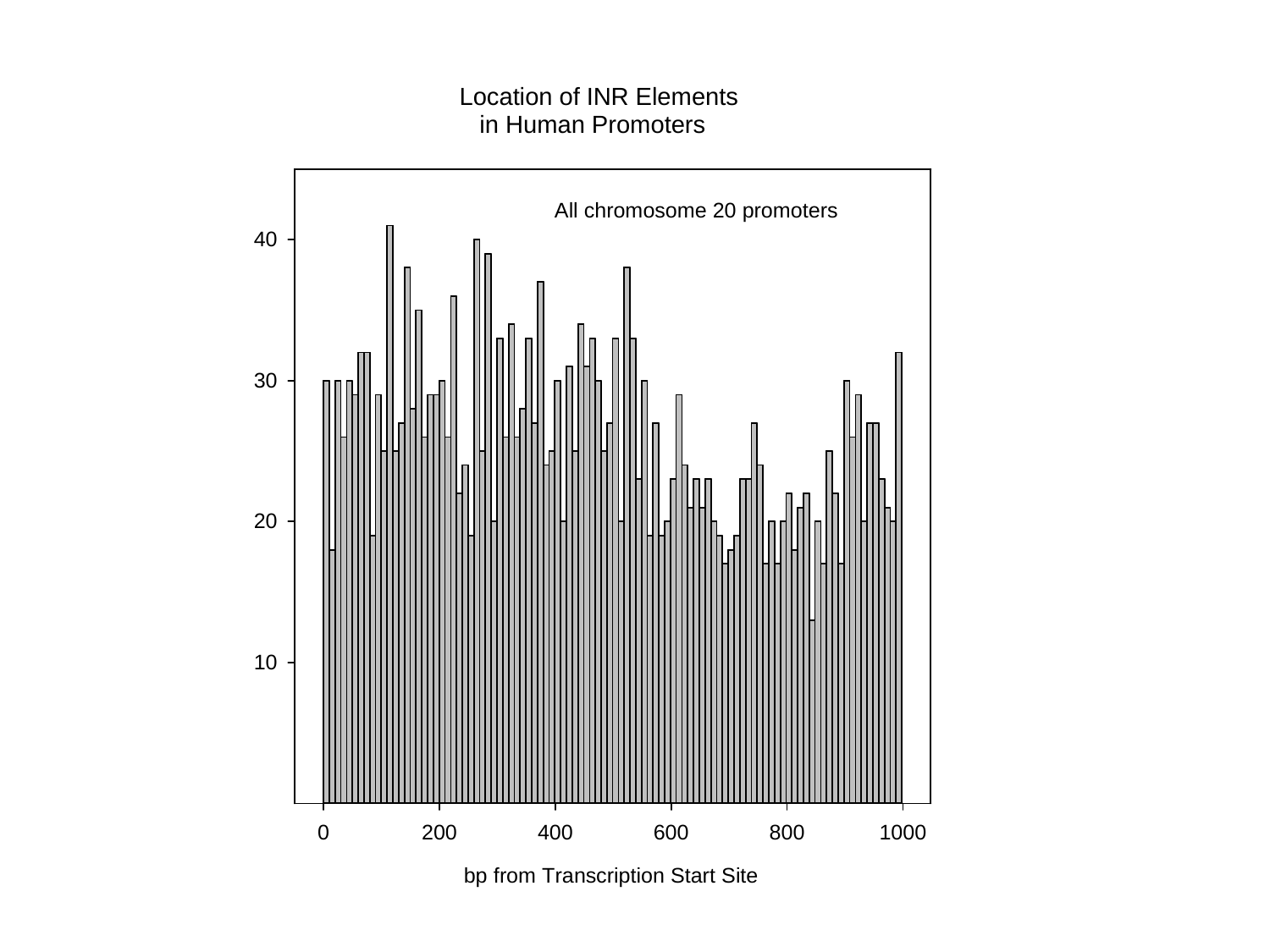

## Slide 22
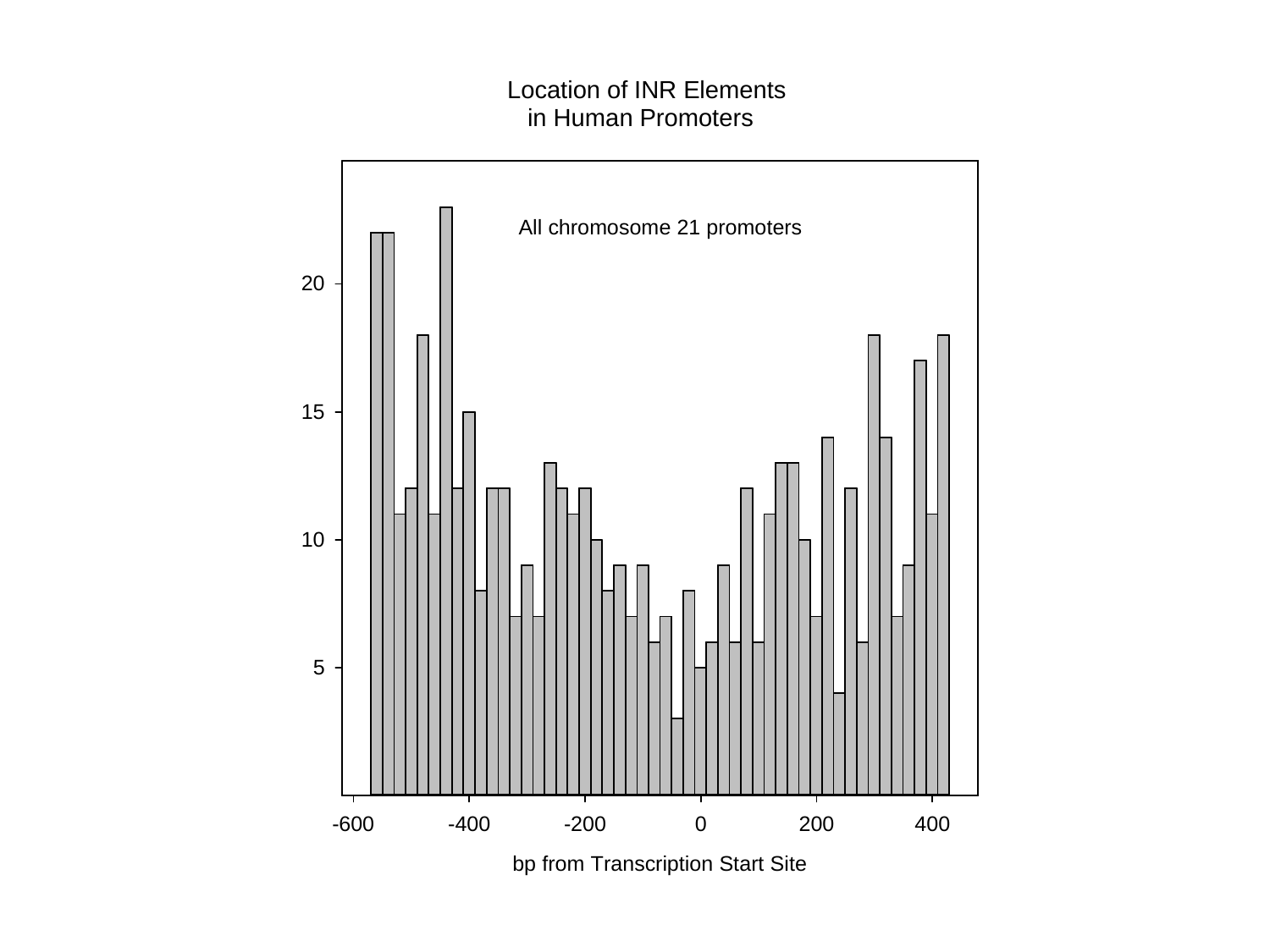

## Slide 23
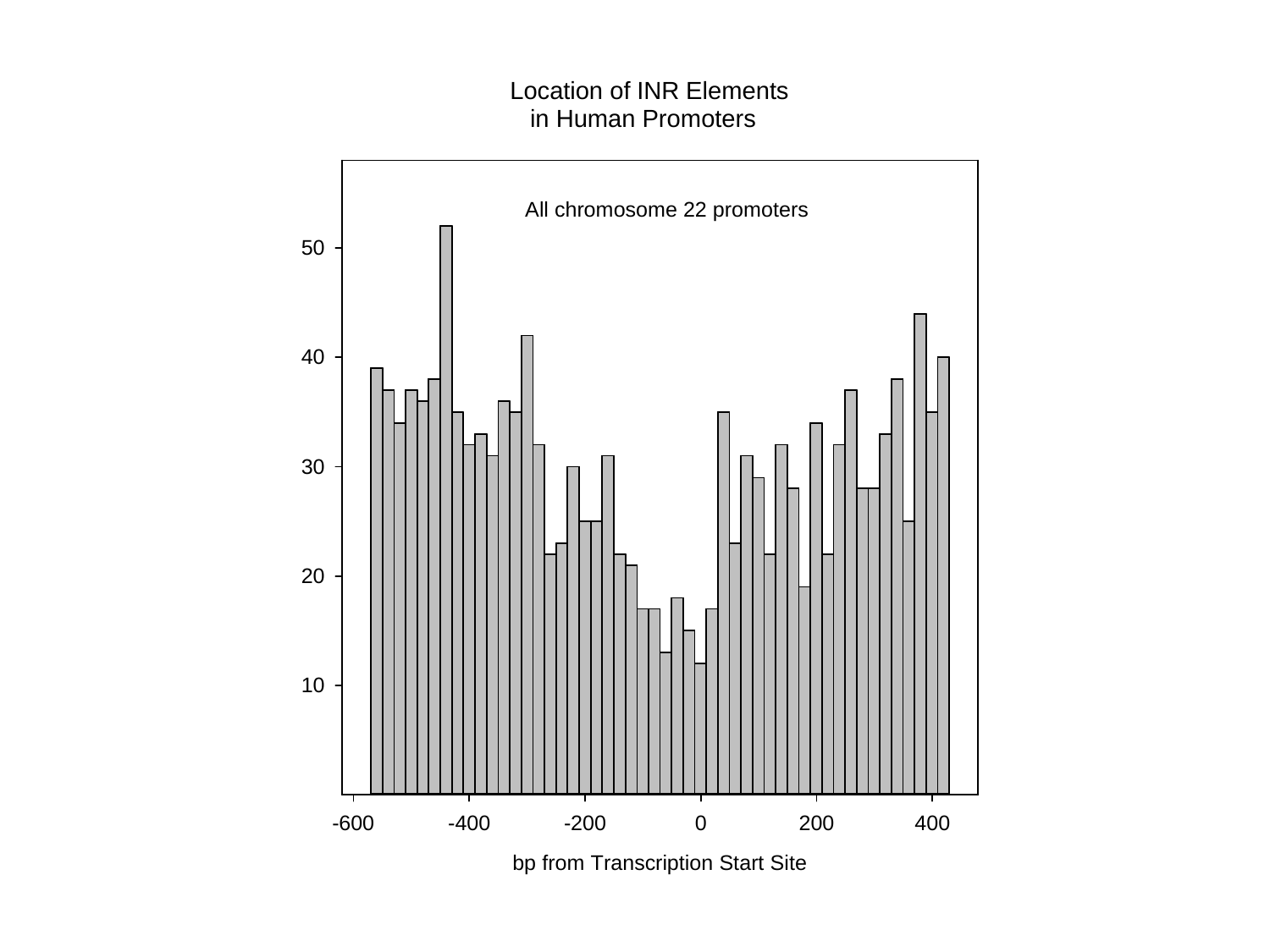

## Slide 24
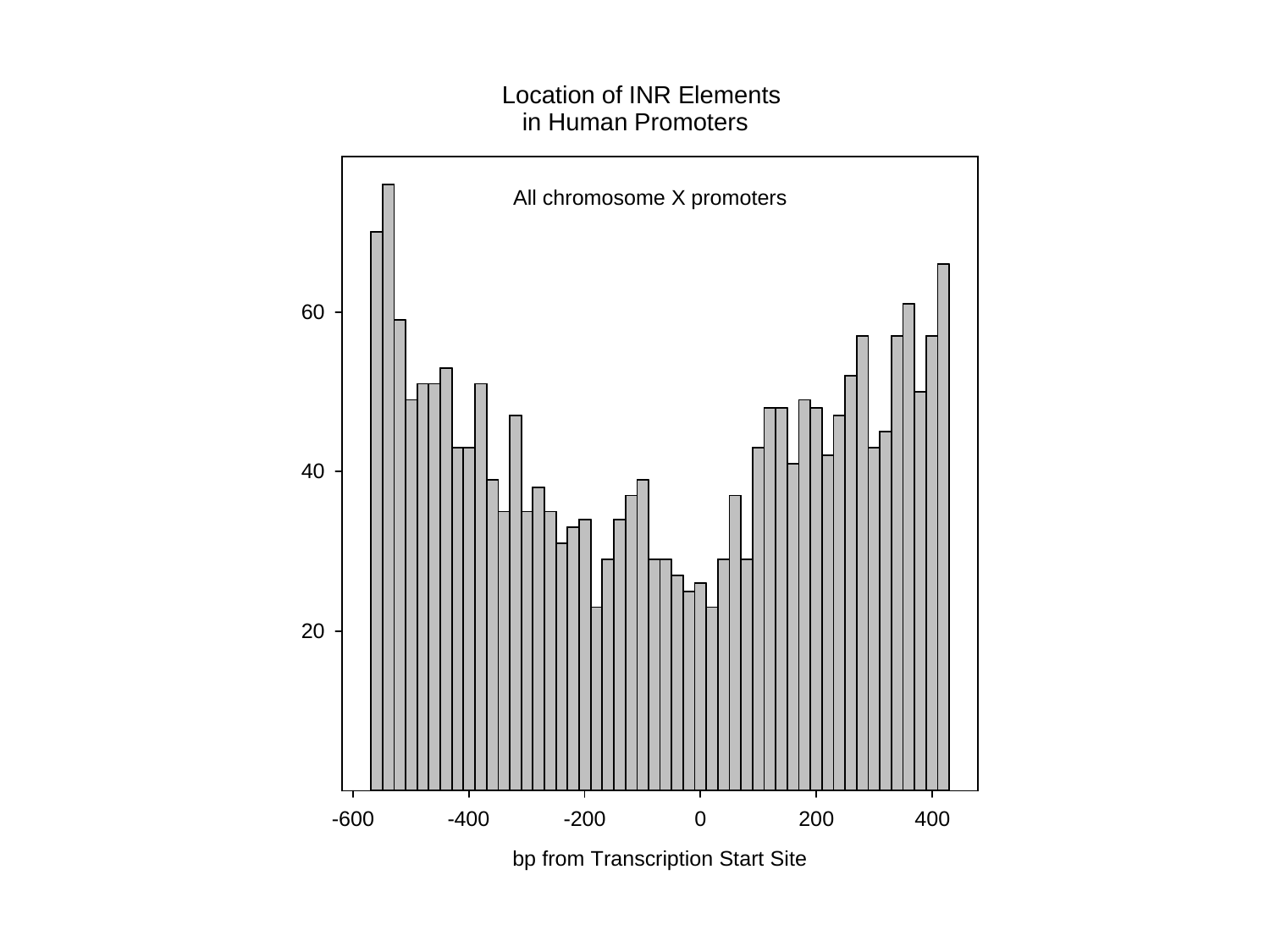

## Slide 25
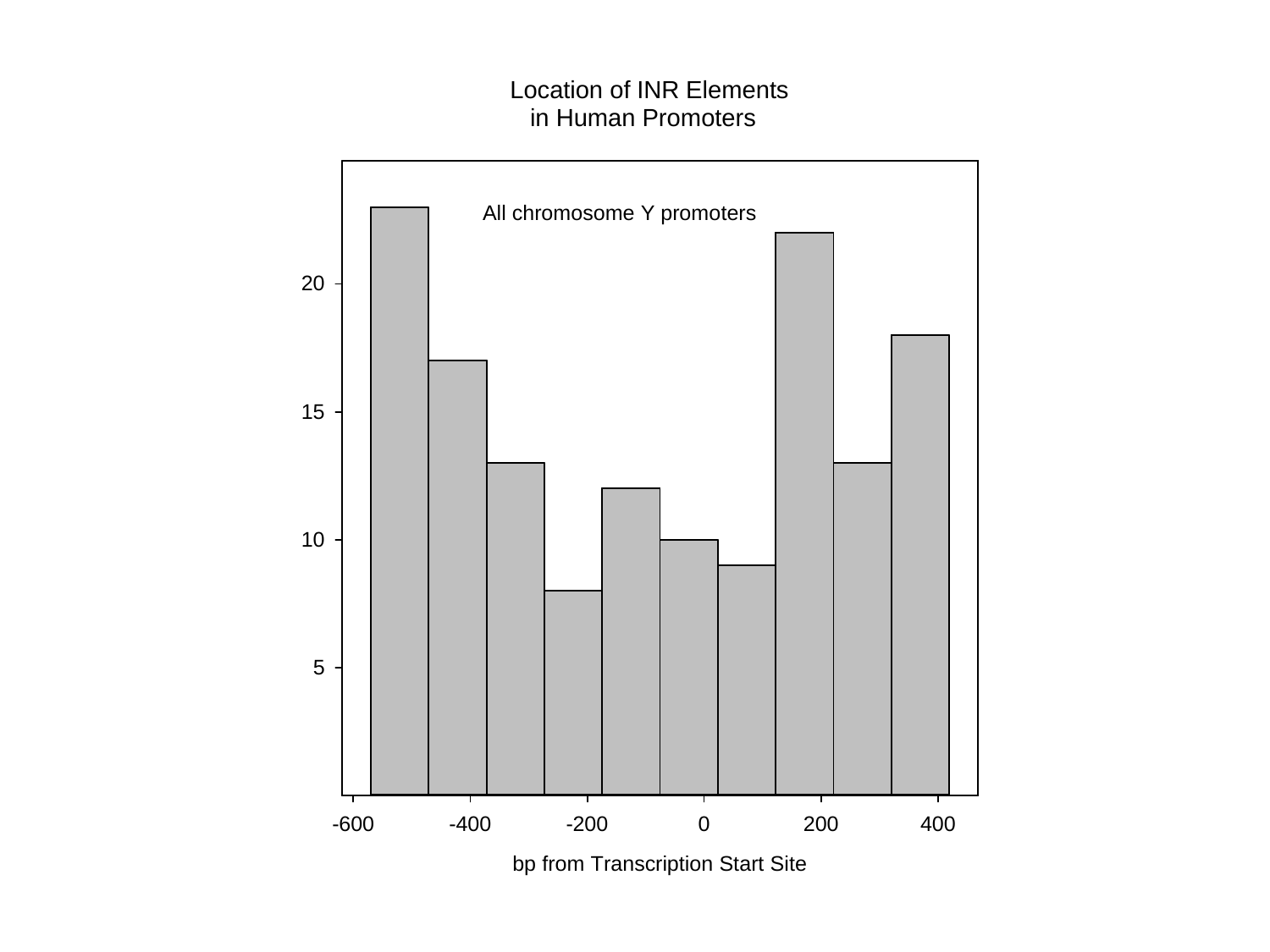

Supplement: S1 Powerpoint — (PPTX) [file pone.0202927.s001.pptx]
