# Supplementary material for: Control of human gene expression: High abundance of divergent transcription in genes containing both INR and BRE elements in the core promoter
Source: PLoS One. 2018 Aug 23;13(8):e0202927. doi: 10.1371/journal.pone.0202927 (PMC6107252; doi:10.1371/journal.pone.0202927)
Supplement: S1 Table — (DOCX) [file pone.0202927.s005.docx]

Supporting Table 1: Divergent Transcription in Human Promoters

Containing both INR and BRE Elements

| Chr | Promoter(s) | R/L/DIV |
| --- | --- | --- |
| 1 | VWA1 / RP4-758J18.10 | DIV |
| 1 | SLC9A1 | L |
| 1 | PUM1 | L |
| 1 | SSBP3 | L |
| 1 | DDAH1 / RP11-131L23.2 | DIV |
| 1 | ATP2B4 | R |
| 1 | AIDA / BROX | DIV |
| 1 | H3F3A / RP11-396C23.4 | DIV |
| 1 | OR2L13 | R |
|  |  |  |
| 2 | WDPCP / MDH1 | DIV |
| 2 | TGFA | L |
| 2 | GCC2-AS1 / LIMS1 | DIV |
| 2 | POLR1B | R |
| 2 | DARS / DARS-AS1 | DIV |
| 2 | NRP2 | R |
| 2 | DUSP28 / ANKMY1 | DIV |
|  |  |  |
| 3 | LHFPL4 | L |
| 3 | FSTL1 | L |
| 3 | PODXL2 | R |
| 3 | KY | L |
| 3 | GHSR | L |
| 3 | FGF12 | L |
|  |  |  |
| 4 | UVSSA | R |
| 4 | PAICS / PPAT | DIV |
| 4 | ANKRD17 / RP11-692D12.1 | DIV |
|  |  |  |
| 5 | HDAC3 / RELL2 | DIV |
|  |  |  |
| 6 | SMAP1 | R |
|  |  |  |
| 7 | FTSJ2 / NUDT1 | DIV |
| 7 | LMTK2 | R |
| 7 | ZC3HAV1L | L |
| 7 | PRKAG2 / PRKAG2-AS1 | DIV |
| 7 | SHH | L |
|  |  |  |
| 8 | ZNF704 | L |
| 8 | RIMS2 / RP11-1C8.4 | DIV |
| 8 | TATDN1 / NOUFB9 | DIV |
| 8 | KHDRBS3 | R |
|  |  |  |
| 9 | DENND4C | R |
| 9 | RORB-AS1 / RORB | DIV |
| 9 | TMEM246 / RNF20 | DIV |
| 9 | NIPSNAP3A | R |
| 9 | PTGR1 | L |
| 9 | ABL1 | R |
| 9 | RP11-251-M1.1 / EGFL7 | DIV |
|  |  |  |
| 10 | VIM-AS1 / VIM | DIV |
| 10 | HNRNPF | L |
| 10 | VSTM4 / FAM170B-AS1 | DIV |
| 10 | NRBF2 / RP11-144G16.1 | DIV |
| 10 | NRAP / CASP7 | DIV |
|  |  |  |
| 11 | RPS13 | L |
| 11 | ACCS | R |
| 11 | PRPF19 / TMEM109 | DIV |
| 11 | TAF6L | R |
| 11 | GAL3ST3 / SF3B2 | DIV |
| 11 | MAP6 | L |
| 11 | PRSS23 | R |
| 11 | NTM | R |
| 11 | THYN1 / ACAD8 | DIV |
|  |  |  |
| 12 | ALG10B | R |
| 12 | RAPGEF3 / SLC48A1 | DIV |
| 12 | RP11-370I10.10 / H1FNT | DIV |
| 12 | CACNB3 | R |
| 12 | NR4A1 | R |
| 12 | DTX3 | R |
| 12 | LINC01465 / RP11-631N16.2 | DIV |
| 12 | GNS / RP11-629N8.3 | DIV |
| 12 | CCT2 | R |
| 12 | PPP1R12A / RP11-84G21.1 | DIV |
| 12 | LIN7A | L |
| 12 | C12orf73 / TDG | DIV |
| 12 | TESC / TESWC-AS1 | DIV |
| 12 | MORN3 | L |
|  |  |  |
| 13 | FARP1 | R |
| 13 | KDELC1 / BIVM | DIV |
|  |  |  |
| 14 | PPP1R3E / /BCL2L2 | DIV |
| 14 | AP1G2 | L |
| 14 | CTD-2540L5.6 / TTC9 | DIV |
| 14 | HEATR4/ ACOT2 | DIV |
| 14 | KCNK13 | R |
| 14 | KLC1 | R |
| 14 | SIVA1 | R |
|  |  |  |
| 15 | LRRC57 / HAUS2 | DIV |
| 15 | GATM / GATM-AS1 | DIV |
| 15 | CYP19A1 / GLDN | DIV |
| 15 | LARP6 / LRRC49 | DIV |
| 15 | CIB2 / IDH3A | DIV |
|  |  |  |
| 16 | ZNF263 | R |
| 16 | ITFG1 / PHKB | DIV |
| 16 | ACD / PARD6A | DIV |
|  |  |  |
| 17 | ALOXE3 | L |
| 17 | STAT5B / STAT5A | DIV |
| 17 | CCR10 / CNTNAP1 | DIV |
| 17 | TEX14 / RAD51C | DIV |
| 17 | CSNK1D | L |
|  |  |  |
| 18 | RP11-49I11.1 / MOCOS | DIV |
| 18 | ACAA2 / SNHG22 | DIV |
| 18 | NETO1 / RP11-676J15.1 | DIV |
| 18 | ATP9B | R |
|  |  |  |
| 19 | FBXL12 / UBL5 | DIV |
| 19 | RAB3D / CCDC159 | DIV |
| 19 | CTD-2369P2.5 / ICAM1 | DIV |
| 19 | AKAP8 / AKAP8L | DIV |
| 19 | CCNE1 | R |
| 19 | LRFN1 | L |
| 19 | RPS16 / SUPT5H | DIV |
| 19 | MIA | R |
| 19 | TMEM145 | R |
|  |  |  |
| 20 | SLC23A2 | L |
| 20 | FERMT1 | L |
| 20 | ZNF341-AS1 / CHMP4B | DIV |
| 20 | C20orf24 | R |
| 20 | MAFB | L |
| 20 | MATN4 / RBPJL | DIV |
| 20 | DIDO1 / GID8 | DIV |
| 20 | CHRNA4 / RP11-261N11.8 | DIV |
|  |  |  |
| 21 | SYNJ1 / PAXBP1-AS1 | DIV |
|  |  |  |
| 22 | RPL3 | L |
| 22 | RRP7A | L |
|  |  |  |
| X | SLC25A6 / LINC00106 | DIV |
| X | ARSD | L |
| X | PIM2 / OTUD5 | DIV |
| X | MAGIX / AC231657.1 | DIV |
|  |  |  |
| Y | SLC25A6 / LINC00106 | DIV |
