# Supplementary material for: Control of human gene expression: High abundance of divergent transcription in genes containing both INR and BRE elements in the core promoter
Source: PLoS One. 2018 Aug 23;13(8):e0202927. doi: 10.1371/journal.pone.0202927 (PMC6107252; doi:10.1371/journal.pone.0202927)
Supplement: S2 Table — (DOCX) [file pone.0202927.s006.docx]

Supporting Table 2: Divergent Transcription in Human Promoters

Containing INR but not BRE Elements

| Chr | Prom | Promoter(s) | R/L/DIV |
| --- | --- | --- | --- |
| 1 | 1004 | CAPN2 | R |
| 1 | 96 | PRAMEF4 | L |
| 1 | 416 | ACOT11 | R |
| 1 | 490 | RPF1 | R |
| 1 | 742 | SMG5 / TMEM79 | DIV |
| 1 | 157 | WNT4 | L |
| 1 | 711 | KCNN3 | L |
| 1 | 447 | DNAJC6 / RP5-1044H5.1 | DIV |
| 1 | 600 | PPM1J / RP11-426L16.9 | DIV |
|  |  |  |  |
| 2 | 549 | ASNSD1 | R |
| 2 | 128 | SPAST | R |
| 2 | 184 | ASB3 / ERLEC1 | DIV |
| 2 | 691 | ARMC9 | R |
| 2 | 647 | CNPPD / FAM134A | DIV |
| 2 | 132 | LTBP1 | R |
| 2 | 584 | PPIL3 / NIF3L1 | DIV |
|  |  |  |  |
| 3 | 499 | COPB2 | L |
| 3 | 203 | RP11-24C3.2 / ATRIP | DIV |
| 3 | 626 | TBCCD1 / DNAJB11 | DIV |
| 3 | 572 | PHC3 | L |
| 3 | 338 | SHQ1 | L |
| 3 | 581 | GHSR | L |
|  |  |  |  |
| 4 | 311 | TIFA | L |
| 4 | 165 | CHIC2 | L |
| 4 | 363 | ZNF330 | R |
|  |  |  |  |
| 5 | 269 | SNCAIP | R |
|  |  |  |  |
| 6 | 235 | CDKN1A | R |
|  |  |  |  |
| 7 | 117 | AC002480.2 | L |
| 7 | 577 | LAMB1 | L |
| 7 | 133 | DFNA5 | L |
| 7 | 718 | CLCN1 | R |
| 7 | 28 | FTSJ2 / NUDT1 | DIV |
|  |  |  |  |
| 8 | 444 | SAMD12 / SAMD12-AS1 | DIV |
| 8 | 316 | ZNF704 | L |
| 8 | 469 | TATDN1 / NDUFB9 | DIV |
| 8 | 388 | RP11-410L14.2 / VPS13B | DIV |
|  |  |  |  |
| 9 | 183 | CNTNAP3P2 pseudo | --------- |
| 9 | 127 | GALT | R |
| 9 | 34 | CD274 | R |
| 9 | 233 | C9orf40 / C9orf40-AS1 | DIV |
| 9 | 638 | REXO4 | L |
| 9 | 387 | EPB41L4B | L |
| 9 | 155 | FAM221B / TMEM8B | DIV |
|  |  |  |  |
| 10 | 342 | NFKB2 | R |
| 10 | 32 | NUDT5 / CDC123 | DIV |
| 10 | 446 | CFAP46 | L |
| 10 | 401 | SFXN4 | L |
| 10 | 128 | VSTM4 | L |
|  |  |  |  |
| 11 | 147 | TSG101 | L |
| 11 | 203 | ACCS | R |
| 11 | 617 | SNX19 | L |
| 11 | 558 | RP11-770J1.5 / ATP5L | DIV |
| 11 | 287 | TAF6L / RP11727F15.11 | DIV |
| 11 | 430 | MAP6 | L |
| 11 | 181 | CSTF3 / CSTF3-AS1 | DIV |
| 11 | 346 | RAB1B / RP11-867G23.2 | DIV |
| 11 | 59 | ART1 / ART5 | DIV |
|  |  |  |  |
| 12 | 804 | POLE / PXMP2 | DIV |
| 12 | 474 | DYRK2 | R |
| 12 | 360 | RP11-753H16.5 | R |
| 12 | 104 | ZCRB1 / PPHLN1 | DIV |
| 12 | 553 | Pseudo | ----------- |
| 12 | 591 | TXNRD1 | R |
| 12 | 665 | OAS3 | R |
| 12 | 390 | RP11-603J24.5 / ESYT1 | DIV |
| 12 | 63 | CIR | L |
| 12 | 660 | NAA25 | L |
| 12 | 175 | ALG10B | R |
| 12 | 68 | SLC2A14 | L |
| 12 | 565 | DEPDC4 / SCYL2 | DIV |
| 12 | 386 | ERBB3 | R |
|  |  |  |  |
| 13 | 220 | KLHL1 / ATXN80S | DIV |
| 13 | 179 | pseudo | ----------- |
|  |  |  |  |
| 14 | 219 | ACOT2 | R |
| 14 | 67 | HECTD1 | L |
| 14 | 273 | SPATA7 | R |
| 14 | 80 | PPP2R3C / KIAAO391 | DIV |
| 14 | 356 | SIVA1 | R |
| 14 | 265 | NRXN3 | R |
| 14 | 209 | CTD-2540L5.6 / TTC9 | DIV |
|  |  |  |  |
| 15 | 289 | CTD-3154N5.1 / CLK3 | DIV |
| 15 | 237 | SNAPC5 | L |
| 15 | 154 | CYP19A1 / GLDN | DIV |
| 15 | 128 | ZSCAN29 / TUBGCP4 | DIV |
| 15 | 283 | CCDC33 | R |
|  |  |  |  |
| 16 | 710 | GSE1 | R |
| 16 | 329 | RP11-345J4.5 / SLX1B | DIV |
| 16 | 575 | ACD / PARD6A | DIV |
|  |  |  |  |
| 17 | 506 | TUBD1 / RPS6KB1 | DIV |
| 17 | 299 | FBXL20 / CTB-131K11.1 | DIV |
| 17 | 254 | C17orf102 / TMEM132E | DIV |
| 17 | 49 | CXCL16 / ZMYND15 | DIV |
| 17 | 4 | GLOD4 / RNMTL1 | DIV |
|  |  |  |  |
| 18 | 165 | ACAA2 / SNHG22 | DIV |
| 18 | 144 | ATP5A1 / HAUS1 | DIV |
| 18 | 223 | RTTN | L |
| 18 | 207 | PIGN / KIAA1468 | DIV |
|  |  |  |  |
| 19 | 681 | CPT1C / CTB-33G10.6 | DIV |
| 19 | 196 | RAVER1 / AC114271.2 | DIV |
| 19 | 753 | ZNF665 / CTD-2245F17.3 | DIV |
| 19 | 511 | C19orf54 / SNRPA | DIV |
| 19 | 2318 | RAB3D | L |
| 19 | 529 | CEACAM7 | L |
| 19 | 310 | HAUS8 / MYO9B | DIV |
| 19 | 418 | RBM42 | R |
|  |  |  |  |
| 20 | 466 | RAE1 | R |
| 20 | 477 | APCDD1L / APCCD1:-AS1 | DIV |
| 20 | 220 | KIF3B | R |
| 20 | 455 | CYP24A1 | L |
| 20 | 567 | RP4-583P15.14 | R |
| 20 | 405 | SLC35C2 / RP11-394O2.3 | DIV |
| 20 | 204 | ID1 | R |
| 20 | 168 | NAPB | L |
|  |  |  |  |
| 21 | 84 | HMGN1 | L |
|  |  |  |  |
| 22 | 178 | APOL3 | L |
| 22 | 268 | MEI1 | R |
|  |  |  |  |
| X | 386 | GABRQ | R |
| X | 199 | NONO | R |
| X | 324 | CT45A2 | L |
| X | 201 | TAF1 | R |
|  |  |  |  |
| Y | 26 | TTTY14 | L |
