# Supplementary material for: Control of human gene expression: High abundance of divergent transcription in genes containing both INR and BRE elements in the core promoter
Source: PLoS One. 2018 Aug 23;13(8):e0202927. doi: 10.1371/journal.pone.0202927 (PMC6107252; doi:10.1371/journal.pone.0202927)
Supplement: S3 Table — (DOCX) [file pone.0202927.s007.docx]

Supporting Table 3: Divergent Transcription in Human Promoters

Containing BRE but not INR Elements

| Chr | Promoter(s) | R/L/DIV |
| --- | --- | --- |
| 1 | LINC00623 | R |
| 1 | AKIRIN1 | R |
| 1 | NOL9 / TASS1R1 | DIV |
| 1 | LHX9 | R |
| 1 | LRRC41 / UQCRH | DIV |
| 1 | IGSF21 | R |
| 1 | FHL3 | L |
| 1 | NUAK2 | L |
| 1 | HLX / HLX-AS1 | DIV |
|  |  |  |
| 2 | IGFBP2 | R |
| 2 | ACVR2A | R |
| 2 | YWHAQ | L |
| 2 | EIF4E2 / TIGD1 | DIV |
| 2 | MEMO1 | L |
| 2 | THNSL2 | R |
| 2 | ATOH8 | R |
|  |  |  |
| 3 | ETV5 | L |
| 3 | ISY1 | L |
| 3 | GTPBP8 | R |
| 3 | KAT2B | R |
| 3 | SLC6A11 | R |
| 3 | PLD1 | L |
|  |  |  |
| 4 | PCGF3 | R |
| 4 | MAML3 | L |
| 4 | SH3RF1 | L |
|  |  |  |
| 5 | MRPS27 / PTCD2 | DIV |
|  |  |  |
| 6 | NOL7 / RP1-223E5.4 | DIV |
|  |  |  |
| 7 | HOXA5 | L |
| 7 | ZNRF2 | R |
| 7 | FTSJ2 / NUDT1 | DIV |
| 7 | BAIAP2L1 | L |
| 7 | TMEM130 | L |
|  |  |  |
| 8 | RP11-115C21.2 / MCPH1 | DIV |
| 8 | PCMTD1 / RP11-110G21.1 | DIV |
| 8 | ASAP1 | L |
| 8 | ZNF7 | R |
|  |  |  |
| 9 | ST6GALNAC6 | L |
| 9 | RASEF | L |
| 9 | PHF19 | L |
| 9 | DMRT3 | R |
| 9 | C9orf50 / NTMT1 | DIV |
| 9 | MED22 / RPL7A | DIV |
| 9 | FAM102A | L |
|  |  |  |
| 10 | GLRX3 | R |
| 10 | VSTM4 | L |
| 10 | FAM213A | R |
| 10 | LOXL4 | L |
| 10 | SUV39H2 | R |
|  |  |  |
| 11 | ANKK1 | R |
| 11 | RDX | L |
| 11 | PACS1 / RP11-1167A19.2 | DIV |
| 11 | MTCH2 | L |
| 11 | ARHGEF12 | R |
| 11 | C11orf63 | R |
| 11 | DDX6 / RP11-158I9.8 | DIV |
| 11 | MRPL23 | R |
| 11 | PKNOX2-AS1 / PKNOX2 | DIV |
|  |  |  |
| 12 | RIC8B / RP11-144F15.1 | DIV |
| 12 | NOS1 | L |
| 12 | RFX4 | R |
| 12 | CCDC64 | R |
| 12 | CCT2 | R |
| 12 | CAPRIN2 / RP11-77I22.2 | DIV |
| 12 | SLC48A1 / RAPGEF3 | DIV |
| 12 | RASSF8 / RASSF8-AS1 | DIV |
| 12 | LIN7A | L |
| 12 | PHB2 / EMG1 | DIV |
| 12 | POLE / PXMP2 | DIV |
| 12 | COL2A1 | L |
| 12 | APAF1 / IKBIP | DIV |
| 12 | DTX1 | R |
|  |  |  |
| 13 | DOCK9 / DOCK9-AS2 | DIV |
| 13 | PDX1-AS1 / PDX1 | DIV |
|  |  |  |
| 14 | CH17-302M23.1 / ABHD4 | DIV |
| 14 | ACOT4 | R |
| 14 | FOXN3 | L |
| 14 | ZBTB25 / ZBTB1 | DIV |
| 14 | ALKBH1 / SLIRP | DIV |
| 14 | CALM1 | R |
| 14 | FERMT2 | L |
|  |  |  |
| 15 | UBE3A | L |
| 15 | HERC1 | L |
| 15 | ZNF592 | R |
| 15 | ST20 / ST20-AS1 | DIV |
| 15 | LCMT2 / ADAL | DIV |
|  |  |  |
| 16 | TOX3 | L |
| 16 | FBXL19-AS1 / FBXL19 | DIV |
| 16 | ABCA3 | L |
|  |  |  |
| 17 | KAT2A / HSPB9 | DIV |
| 17 | AMZ2P1 | L |
| 17 | ABCC3 | R |
| 17 | YPEL2 | R |
| 17 | HS3ST3A1 | L |
|  |  |  |
| 18 | NETO1 / RP11-676J15.1 | DIV |
| 18 | RP11-699A5.2 / GREB1L | DIV |
| 18 | RP11-806L2.5 / TYMS | DIV |
| 18 | RP13-270P17.1 / MYL12B | DIV |
|  |  |  |
| 19 | TTYH1 | R |
| 19 | OLFM2 | L |
| 19 | TLE2 | L |
| 19 | HOMER3 / AC005932.1 | DIV |
| 19 | COMP | L |
| 19 | KPTN / NAPA-AS1 | DIV |
| 19 | FSTL3 | R |
| 19 | TMEM145 | R |
|  |  |  |
| 20 | RP5-858L17.1 / SIRPA | DIV |
| 20 | CEBPB-AS1 / CEBPB | DIV |
| 20 | SYCP2 / FAM217B | DIV |
| 20 | RSPO4 | L |
| 20 | RAB22A / PPP4R1L | DIV |
| 20 | NFATC2 | L |
| 20 | CHD6 | L |
| 20 | RP4-738P15.1 | L |
|  |  |  |
| 21 | C21orf33 | R |
|  |  |  |
| 22 | ATXN10 | R |
| 22 | pseudo | ----------- |
|  |  |  |
| X | CETN2 /NSDHL | DIV |
| X | MTMR1 | R |
| X | FUNDC2 | R |
| X | CXorf38 | L |
|  |  |  |
| Y | TBL1Y | R |
