# Supplementary material for: Control of human gene expression: High abundance of divergent transcription in genes containing both INR and BRE elements in the core promoter
Source: PLoS One. 2018 Aug 23;13(8):e0202927. doi: 10.1371/journal.pone.0202927 (PMC6107252; doi:10.1371/journal.pone.0202927)
Supplement: S4 Table — (DOCX) [file pone.0202927.s008.docx]

Supporting Table 4: Divergent Transcription in Randomly

Selected Human Promoters

| Chr | Promoter(s) | R/L/DIV |
| --- | --- | --- |
| 1 | SASS6 / TRMT13 | DIV |
| 1 | HCRTR1 | R |
| 1 | UHMK1 | R |
| 1 | RCOR3 | R |
| 1 | UBE2J2 / RP5-902P8.10 | DIV |
| 1 | EFHD2 / RP3-467K16.4 | DIV |
| 1 | OPN3 | L |
| 1 | SLC35F3 | R |
| 1 | GUK1 | R |
|  |  |  |
| 2 | THAP4 / ATG4B | DIV |
| 2 | TMEM163 | L |
| 2 | ACVR1C | L |
| 2 | REEP1 | L |
| 2 | ALMS1 | R |
| 2 | TSGA10 / C2orf15 | DIV |
| 2 | WDR12 / CARF | DIV |
|  |  |  |
| 3 | B3GALNT1 | L |
| 3 | ABCF3 | R |
| 3 | RAB6B | L |
| 3 | ROPN1B | R |
| 3 | ASTE1 / NEK11 | DIV |
| 3 | SH3BP5 | L |
|  |  |  |
| 4 | C4orf32 | R |
| 4 | LETM1 | L |
| 4 | CRMP1 | L |
|  |  |  |
| 5 | C5orf45 / CTC-241N9.1 | DIV |
|  |  |  |
| 6 | HMGN4 | R |
|  |  |  |
| 7 | STEAP4 | L |
| 7 | CLEC2L | R |
| 7 | IQUB | L |
| 7 | NSUN5 | L |
| 7 | SNX8 | L |
|  |  |  |
| 8 | PHF20L1 | R |
| 8 | KHDRBS3 | R |
| 8 | LOXL2 | L |
| 8 | SHARPIN / MAF1 | DIV |
|  |  |  |
| 9 | SEC16A / C9orf63 | DIV |
| 9 | TMEFF1 | R |
| 9 | KLF9 | L |
| 9 | MOB3B | L |
| 9 | CEP78 | R |
| 9 | TLE1 / RP11-154D17.1 | DIV |
| 9 | GKAP1 | L |
|  |  |  |
| 10 | RRR12 / PGAM1 | DIV |
| 10 | SLC16A12 | L |
| 10 | KIN / ATP5C1 | DIV |
| 10 | ASCC1 / ANAPC16 | DIV |
| 10 | CHST15 | L |
|  |  |  |
| 11 | ART5 | L |
| 11 | DPF2 | R |
| 11 | NAV2 | R |
| 11 | IFITM1 | R |
| 11 | TPCN2 | R |
| 11 | PIH1D2 / C11orf57 | DIV |
| 11 | RP11-613D13.5 / ALKBH3 | DIV |
| 11 | EIF3M | R |
| 11 | GANAB | L |
|  |  |  |
| 12 | RP11-415I12.3 / SRGAP1 | DIV |
| 12 | ALDH2 | R |
| 12 | FGFR1OP2 / ASUN | DIV |
| 12 | STAT2 | L |
| 12 | SYCP3 | L |
| 12 | GIT2 / ANKRD13A | DIV |
| 12 | NECAP1 | R |
| 12 | MAP3K12 / TARBP2 | DIV |
| 12 | UBE2N | L |
| 12 | PMEL / CDK2 | DIV |
| 12 | METAP2 | R |
| 12 | TESC / TESC-AS1 | DIV |
| 12 | YARS2 | L |
| 12 | SENP1 / PFKM | DIV |
|  |  |  |
| 13 | C1QTNF9 | R |
| 13 | ENOX1 | L |
|  |  |  |
| 14 | NIN | L |
| 14 | EIF2B2 | R |
| 14 | REC8 | R |
| 14 | TMED10 | L |
| 14 | BCL11B | L |
| 14 | VIPASS9 / AHSA1 | DIV |
| 14 | CKB | L |
|  |  |  |
| 15 | NDNL2 | L |
| 15 | SHC4 | L |
| 15 | HCN4 | L |
| 15 | SH3GL3 | R |
| 15 | MPI | R |
|  |  |  |
| 16 | ADCY9 | L |
| 16 | ERI2 / AC004381.6 | DIV |
| 16 | CHTF8 / CIRH1A | DIV |
|  |  |  |
| 17 | ERN1 | L |
| 17 | CBX2 | R |
| 17 | TMEM92 | R |
| 17 | COA3 / CNTD1 | DIV |
| 17 | SPOP | L |
|  |  |  |
| 18 | CLUL1 | R |
| 18 | RP11-162A12.2 /ZNF236 | DIV |
| 18 | NOL4 / RP11-379L18.1 | DIV |
| 18 | RPRD1A | L |
|  |  |  |
| 19 | ZNF432 | L |
| 19 | PRTN3 | R |
| 19 | GNG7 | L |
| 19 | AKAP8 / AC005785.2 | DIV |
| 19 | LIPE-AS1 | R |
| 19 | AXL | R |
| 19 | ZFP82 | L |
| 19 | TTYH1 | R |
|  |  |  |
| 20 | NRSN2-AS1 / NRSN2 | DIV |
| 20 | ZNFX1 / ZFAS1 | DIV |
| 20 | GATA5 | L |
| 20 | MAVS | R |
| 20 | MATN4 / RBPJL | DIV |
| 20 | AP5S1 | R |
| 20 | SAMD10 / PRPF6 | DIV |
| 20 | GPCPD1 | L |
|  |  |  |
| 21 | CRYZL1 / ITSN1 | DIV |
|  |  |  |
| 22 | PLA2G6 | L |
| 22 | NAGA | L |
|  |  |  |
| X | SPANXA1 | L |
| X | TMEM255A | L |
| X | PCDH19 | L |
| X | SLITRK4 | L |
|  |  |  |
| Y | USP9Y | R |
